# Waste-Derived Natural Polymer Biosorbents for Toxic Metal Ions: Structure–Sorption Relationships, Modification Strategies, and Prospects for Solid-Phase Sensing

**DOI:** 10.3390/polym18192336

**Published:** 2026-09-24

**Authors:** Aisha Nurlybayeva, Zulaykho Smanova, Zhanna Tazhiyeva, Zulfiya Unerbayeva, Raikhan Rakhmetullayeva, Gulsim Matniyazova, Ainur Seitkhan, Raushan Taubayeva, Bekzat Saurbayeva, Aitkul Aidarova, Ergali Rustem

**Affiliations:** 1 Department of Chemistry and Chemical Technology, M.Kh. Dulaty Taraz University, Taraz 080000, Kazakhstan; rustem_ergali@mail.ru (A.N.); ao.aidarova@dulaty.kz (A.A.); 2 Department of Analytical Chemistry, Faculty of Chemistry, National University of Uzbekistan, Tashkent 100174, Uzbekistan; smanova.chem@mail.ru; 3 Department of Chemistry, Kazakh National Pedagogical University named after Abai, Almaty 050010, Kazakhstan; uner_68@mail.ru; 4 Department of Chemistry and Technology of Organic Substances, Natural Compounds and Polymers, Faculty of Chemistry and Chemical Technology, Al-Farabi Kazakh National University, Almaty 050040, Kazakhstan; raikhan.rakhmetullayeva@gmail.com; 5 Higher School of Natural Sciences, Astana International University, Astana 010000, Kazakhstan; gulsim.matniyazova@mail.ru; 6 Department of Environmental Management and Engineering, L.N. Gumilyov Eurasian National University, Astana 010008, Kazakhstan; seitkan_as@enu.kz; 7 School of International Engineering, D. Serikbayev East Kazakhstan Technical University, Ust-Kamenogorsk 070004, Kazakhstan; saurbaeva71@mail.ru; 8 Faculty of Architecture, Construction and Transport, Mukhtar Auezov South Kazakhstan University, Shymkent 160012, Kazakhstan; rustem-ergali79@mail.ru

**Keywords:** waste-derived biosorbents, silk fibroin, banana peel, orange peel, pectin, toxic metal ions, heavy metals, adsorption, regeneration, diffuse reflectance

## Abstract

Waste-derived natural polymers offer a route to low-cost sorbents for toxic metal ions, but reported performance is difficult to compare because precursor composition, polymer processing, modification, and experimental conditions vary widely. This critical review examines silk fibroin, banana peel, orange peel, and pectin as structurally distinct platforms and evaluates how macromolecular chemistry, esterification, ionic crosslinking, morphology, and regeneration influence metal uptake. Particular attention is given to the distinction between untreated biomass, isolated biopolymers, and chemically engineered materials; to mechanistic evidence from spectroscopy, elemental analysis, ion exchange, and controlled structural modification; and to the limitations of interpreting Langmuir or pseudo-second order fitting as direct proof of adsorption mechanism. The literature supports strong roles for pectic carboxyl groups and Ca^2+^-mediated exchange in several cation-binding systems, while silk fibroin provides a reusable protein-based matrix with different donor-site chemistry. Across the four platforms, high q_max_ values are not transferable material constants and do not by themselves establish selectivity, practical treatment performance, or analytical sensitivity. Major gaps include precursor standardization, matched comparison of raw and modified sorbents, multicomponent and real-water testing, nonlinear model evaluation, continuous-flow studies, regeneration stability, metal recovery, and end-of-life management. Diffuse-reflectance sensing is considered as a downstream value-added function once a reproducible biosorbent has been established. An evidence-based roadmap is proposed for converting polymer-rich wastes into controlled, regenerable, and application-oriented biosorbents.

## 1. Introduction

Contamination of natural and industrial waters with potentially toxic metal ions remains a persistent environmental problem because metals are not chemically degraded and may accumulate in sediments and biological systems. Pb(II), Cd(II), Cu(II), Ni(II), and chromium species are among the ions most frequently considered in water-treatment studies. Instrumental techniques such as AAS, ICP-OES, and ICP-MS remain indispensable for accurate determination, but remediation itself requires materials capable of capturing dissolved metal species efficiently, selectively, and preferably with low material and processing costs.

This requirement has sustained interest in biosorbents derived from renewable resources and agro-industrial residues. Natural polymer-rich wastes are attractive because their macromolecular components contain carboxyl, hydroxyl, carbonyl, amino, and other polar groups that can participate in ion exchange, complexation, electrostatic interactions, and hydrogen-bond-assisted surface organization [1]. Their value, however, lies not simply in being inexpensive or biologically derived. Sorption performance depends on the accessibility and chemical state of these groups, polymer-chain organization, porosity, swelling, particle size, crosslinking, and the processing history of the precursor. For this reason, a useful comparison must connect waste composition with polymer structure and subsequently with metal-binding behavior.

The present review focuses on four related but structurally distinct platforms: silk fibroin obtained from silk-processing residues, banana peel, orange peel, and pectin as the principal controllable anionic polymer that can be isolated from pectin-rich plant wastes. Banana and orange peels are heterogeneous cell-wall matrices containing pectic substances together with cellulose, hemicellulose, lignin, and minor constituents. Their ability to bind Cu (II), Co(II), Ni(II), Zn(II), Pb(II), Cd(II), and chromium species has been demonstrated in untreated and modified forms [2,3]. Banana peel is also a source of extractable pectin, with reported extraction yield and degree of esterification varying with botanical variety and processing conditions [4].

Pectin is considered separately because isolation and controlled modification make it possible to examine structure–property relationships that are difficult to resolve in whole fruit peel. Its galacturonic-acid-rich backbone contains carboxyl groups whose ionization and availability are governed by pH, degree and distribution of esterification, and ionic crosslinking. In Ca-crosslinked pectin gels, displacement of Ca(II) and interaction of incoming metals with carboxylate groups provide direct evidence for ion-exchange and coordination contributions to Cd(II), Pb(II), and Cu(II) uptake [5]. Processing pectin into films, foams, gels, xerogels, and microspheres further demonstrates that morphology and network architecture can be varied independently of precursor identity [6].

Silk fibroin represents a complementary protein-based platform. Its semicrystalline polypeptide structure differs fundamentally from the polysaccharide-rich plant matrices and provides peptide carbonyls, hydroxyl-bearing residues, and a smaller population of ionizable side and terminal groups. Fibroin obtained from *Bombyx mori* has been investigated for Pb(II), chromate, Cu(II), Co(II), and Fe removal, and its ability to withstand repeated sorption–regeneration cycles is particularly relevant when a waste-derived material is intended for reuse [7]. Thus, the four platforms span a useful range from chemically heterogeneous biomass to isolated polysaccharide and semicrystalline protein matrices.

A major difficulty in the existing literature is that adsorption capacities are often compared without adequate separation of untreated biomass, isolated polymers, chemically modified materials, and composite networks. Extraction can expose previously inaccessible sites; demethylation increases the fraction of free pectic carboxyl groups; Ca(II) crosslinking introduces exchangeable counterions and changes network structure; grafting can create entirely new binding functionalities; and freeze-drying or other processing can substantially alter porosity. Consequently, the highest reported q_max_ is not necessarily evidence that one waste precursor is intrinsically superior to another. Critical interpretation requires the material form and preparation history to remain attached to every quantitative value.

Mechanistic interpretation presents a related problem. Good fits to Langmuir, Freundlich, pseudo-first-order, or pseudo-second-order equations are useful descriptors but do not independently identify molecular binding pathways. Greater evidential weight should be assigned to studies combining adsorption measurements with FTIR, SEM–EDX, XPS, ion-release measurements, zeta potential, controlled esterification, or matched comparison of native and depectinated/crosslinked materials. This distinction is central to the present review because rational biosorbent design requires knowledge of which structural changes create or expose effective binding sites.

Regeneration and practical reuse are equally important. A material that removes a large amount of metal in its first cycle may still be unsuitable if desorption damages the polymer network, if capacity declines rapidly, or if regeneration requires large quantities of hazardous reagents. The environmental benefit of a waste-derived biosorbent must therefore be assessed over the complete use cycle, including preparation, sorption, desorption, regeneration, metal recovery, and management of the spent metal-containing phase. Natural origin alone is not sufficient to establish analytical or environmental sustainability [8].

A secondary but potentially valuable opportunity is to convert a well-characterized biosorbent into a functional sensing phase. Most adsorption studies determine the remaining metal concentration in solution and do not ask whether metal loading produces a reproducible optical response in the solid material. Diffuse-reflectance methods are attractive for opaque powders, fibers, films, and porous polymer matrices, and pectin chemistry can already be quantified in the solid state by DRIFTS [9,10]. Nevertheless, sorption capacity and analytical sensitivity are different properties. Solid-phase sensing is therefore treated here as a downstream functional prospect rather than as the primary criterion for selecting a biosorbent.

This emphasis also distinguishes the present review from broader syntheses of pectin-based metal adsorbents and pectin surface chemistry [11,12]. Recent work has shown that pectin source, extraction route, molecular structure, and material format strongly affect downstream performance [13], while reviews of silk-fibroin composites demonstrate that functionalization can markedly enhance metal uptake [14]. These bodies of literature are valuable, but they also show why native, minimally processed, and strongly engineered materials must not be conflated.

Existing reviews have generally treated pectin-based adsorbents, pectin surface chemistry, silk-fibroin composites, or broader natural-polymer remediation as separate studies [11,12,13,14]. The present review addresses a different comparison problem: it follows the same evidence chain across chemically heterogeneous waste biomass, isolated natural polymers, and deliberately engineered polymer networks while keeping material class and preparation history attached to every quantitative value. It further integrates selectivity, regeneration stability, real-water and continuous-flow translation, and optional solid-phase sensing within a single structure–property–performance framework. This cross-class, source-traceable comparison is the principal rationale for a further review rather than a repetition of existing material-specific surveys.

Historically, the field progressed from direct use of minimally processed agro-wastes toward increasingly controlled polymer materials. Early studies established that raw banana and orange peels could remove several divalent metals and showed strong effects of pH, particle size, and surface charge [2,3]. At that stage, adsorption capacity and isotherm fitting were often the principal performance descriptors, and the heterogeneous peel was commonly treated as a single biosorbent material rather than as a multicomponent polymer matrix.

A second phase shifted attention toward the molecular origin of binding sites. Isolation of pectin, comparison of esterification states, depectination experiments, and Ca^2+^-crosslinked pectin gels demonstrated that accessible galacturonate carboxyl groups and exchangeable counterions can be tested directly rather than inferred from whole-biomass composition [5,11,12]. In parallel, controlled processing of silk fibroin and chemically modified fruit-peel matrices showed that polymer morphology, network architecture, and newly introduced functional groups can change both uptake and reusability [7,14].

Several early conclusions remain robust: solution pH strongly controls ionization and metal speciation; carboxyl-rich domains are important for many cationic metals; and precursor processing changes apparent performance. Other interpretations now require qualification. A high model-derived q_max_ should not be treated as an intrinsic material constant, pseudo-second-order fitting is not independent proof of chemisorption, and values obtained for raw biomass, isolated polymer, and strongly engineered networks should not be pooled as if they represented the same material class. The present review uses this historical transition to define which evidence remains transferable and which earlier simplifications should no longer guide material selection.

Accordingly, the principal objective of this review is to establish design-relevant relationships among precursor composition, polymer structure, modification strategy, metal-ion sorption, selectivity, and regeneration for silk fibroin, banana peel, orange peel, and pectin-based materials. A second objective is to identify which material–metal combinations have sufficient structural and mechanistic support to justify further development, including optional solid-phase optical sensing. The review therefore asks not which published material has the single highest adsorption capacity, but which structural features and processing choices provide the most defensible basis for developing reproducible, reusable, waste-derived biosorbents for toxic metal ions.

## 2. Literature Search Strategy and Review Scope

This article was designed as a critical narrative review supported by a structured literature search rather than as a systematic review or meta-analysis. The aim is to integrate evidence from heterogeneous materials and experimental traditions while retaining sufficient source traceability for quantitative adsorption and analytical data. A systematic-review label would require a fully reproducible protocol and formal accounting of all retrieved and excluded records [15,16].

The literature search was organized around four connected questions: (i) how the macromolecular structure and composition of silk fibroin, banana peel, orange peel, and pectin control metal-ion binding; (ii) how extraction, demethylation, ionic crosslinking, grafting, particle-size control, and other modifications change sorption behavior; (iii) how pH, metal speciation, competing ions, regeneration, and material format affect the practical performance of these biosorbents; and (iv) whether any of the resulting materials show a credible pathway toward direct solid-phase optical sensing as an additional function.

The literature search was updated through August 2026. Relevant publications were identified using PubMed and major publisher platforms, including ScienceDirect, Wiley Online Library, MDPI, RSC Publishing, and SpringerLink, together with DOI-based cross-checking and backward citation tracking from key primary studies and recent reviews. The search was designed to provide broad coverage of the selected polymeric biosorbents, their structural characteristics, modification strategies, metal-ion sorption behavior, regeneration, and potential analytical applications. Priority was given to peer-reviewed primary studies providing experimentally supported adsorption, structural, mechanistic, regeneration, or analytical data, while recent reviews were used to contextualize the state of the field and identify research gaps.

During revision, a targeted update extended the search through September 2026, with emphasis on 2026 primary studies addressing polymer design, adsorption/removal mechanisms, regeneration, multicomponent performance, and practical water-treatment validation. Newly identified studies were used to update the design discussion without pooling them quantitatively with materially different systems.

Material-related search terms included “silk fibroin”, “silk processing waste”, “*Bombyx mori* fibroin”, “banana peel”, “orange peel”, “citrus peel”, “pectin”, “pectic acid”, “calcium pectate”, “pectin gel”, “pectin hydrogel”, and “pectin microsphere”. These terms were combined with metal names and oxidation states (Pb(II), Cd(II), Cr(VI), Cr(III), Cu(II), Ni(II), Mn(II), Co(II), Zn(II)) and with process terms including “adsorption”, “biosorption”, “ion exchange”, “complexation”, “crosslinking”, “demethylation”, “regeneration”, “desorption”, “selectivity”, “degree of esterification”, “diffuse reflectance”, “DRIFTS”, “Kubelka–Munk”, and “solid-phase spectrophotometry”.

Priority was given to peer-reviewed primary studies reporting precursor preparation, material modification, solution conditions, adsorption performance, regeneration, or analytical figures of merit. Studies containing FTIR, SEM–EDX, XPS, zeta-potential, ion-release, controlled esterification, depectination, or crosslinking data were assigned greater mechanistic weight than studies in which mechanism was inferred only from equilibrium or kinetic fitting. Recent reviews were used to frame the state of the field and identify gaps, but numerical adsorption values were preferentially traced to primary articles [11,12].

Studies were not pooled quantitatively when the precursor had been fundamentally transformed. Carbonized fruit-peel biochars and activated carbons were excluded from direct comparison with native polymer-rich biomass. Grafted, mineral-loaded, activated–carbon-containing, strongly acid/alkali-treated, and ionically crosslinked materials were retained only when they clarified how deliberate material engineering changes adsorption, regeneration, or sensing behavior, and they are explicitly separated from untreated materials. This distinction is necessary because modification can alter functional-group density, porosity, swelling, diffusion, and the chemical identity of the binding sites [11,12].

For adsorption data, reported values are retained with the corresponding material form and experimental context. Langmuir q_max_, experimentally measured uptake, removal percentage, affinity constants, and analytical detection limits are treated as different quantities and are not interchanged. Agreement with pseudo-second-order kinetics is not accepted as proof of chemisorption without independent structural or chemical evidence. For the optional sensing perspective, the review distinguishes adsorption followed by solution-phase determination, immobilized-reagent solid-phase spectrophotometry, and a reagent-free optical response of the polymer matrix itself.

## 3. Polymer Chemistry and Material Characteristics of the Selected Biosorbent Precursors

The four precursor classes considered in this review differ not only in biological origin but, more importantly, in the chemical organization of their polymer matrices. Silk fibroin is a protein-based semicrystalline macromolecule, whereas banana and orange peels are heterogeneous plant-cell-wall residues containing several classes of polysaccharides and lignin. Pectin, when isolated from plant waste, represents a more chemically defined anionic polysaccharide in which the density and distribution of ionizable carboxyl groups can be deliberately altered through esterification state and ionic crosslinking. These structural differences govern hydration, ion diffusion, accessibility of binding sites, mechanical stability, regeneration, and the response to chemical modification.

For this reason, comparison of the selected precursors solely based on maximum adsorption capacity is insufficient. A high q_max_ may reflect many sites made accessible by a particular preparation route, but it does not by itself identify the chemical nature of those sites, their selectivity, or their stability during reuse. A structure-based comparison is therefore required before adsorption data can be used to select a waste precursor or modification strategy for biosorbent development. The comparative structural features of the four selected biosorbent precursor platforms are summarized in Figure 1.

### 3.1. Silk Fibroin: A Semicrystalline Protein Matrix

Silk fibroin from *Bombyx mori* has a well-defined hierarchical organization that distinguishes it from the polysaccharide-based materials considered below. Native fibroin contains a heavy chain of approximately 390 kDa and a light chain of approximately 26 kDa linked through a disulfide bond; the complex is associated non-covalently with the P25 glycoprotein. The heavy chain is dominated by glycine, alanine, and serine and contains repetitive Gly–X sequences, including characteristic Gly/Ala/Ser-rich motifs [19,20]. These repetitive segments favor the formation of ordered β-sheet-rich domains, whereas less repetitive and more polar sequences occur predominantly in the amorphous regions of the protein.

This crystalline–amorphous organization is relevant to metal sorption. The densely packed β-sheet regions provide structural stability but are not necessarily the most accessible regions for ion binding. By contrast, peptide carbonyl groups and polar amino-acid residues located at hydrated surfaces and within less ordered domains are more readily exposed to the aqueous phase. Serine and tyrosine provide hydroxyl-containing side chains, while carboxyl- and amino-containing residues, although present in substantially lower proportions than glycine and alanine, contribute additional ionizable sites. The peptide backbone itself supplies a high density of carbonyl oxygen atoms. Consequently, the chemical environment available for metal interaction is heterogeneous even though fibroin is a single biopolymer.

This point requires some caution in describing the sorption mechanism. Fibroin is sometimes represented schematically as a polymer rich in free amino groups; however, this description overstates their abundance in *Bombyx mori* fibroin. The major repeated sequences are dominated by Gly, Ala, and Ser, and most nitrogen atoms are incorporated into peptide bonds rather than present as primary amines. A more realistic description is therefore that metal uptake may involve the collective contribution of peptide carbonyls, hydroxyl-bearing residues, ionizable side groups, terminal groups, and the electrostatic environment of the hydrated protein surface. Direct assignment of a single coordination geometry would require spectroscopic evidence specific to the metal–fibroin pair.

Huang et al. provided the most relevant experimental evidence for fibroin among the studies considered in this review [7]. Fibroin extracted from *Bombyx mori* cocoons was shown to sorb Pb(II), chromate, Cu(II), and Co(II) from aqueous solutions. Thermodynamic analysis indicated spontaneous and endothermic sorption under the conditions investigated, while the authors classified the process as physical sorption. Of particular importance for the present discussion, the metal-loaded fibroin could be treated with EDTA and reused over ten sorption–regeneration cycles without a measurable loss of sorption capacity [7]. This result provides stronger evidence for the structural stability of fibroin as a reusable sorption phase than for any specific coordination mechanism.

The distinction is important for reusable biosorbent design. A mechanically and chemically stable protein can potentially tolerate repeated loading, washing, desorption, and regeneration, but retention of bulk capacity should be accompanied by evidence that the polymer structure and accessible surface remain reproducible. Where the same material is later evaluated as a sensing phase, changes in hydration, β-sheet content, roughness, or packing would also need to be controlled because they can modify optical scattering independently of sorption capacity.

The structural interpretation adopted here is consistent with the wider silk-polymer literature. Vepari and Kaplan described silk fibroin as a processable protein polymer whose properties can be altered through amino-acid side-chain chemistry [17], while Rockwood et al. demonstrated that regenerated *Bombyx mori* fibroin can be reproducibly fabricated as films, fibers, hydrogels, sponges, microspheres, and other morphologies [18]. These studies are important for biosorbent design because the same chemical composition can be processed into morphologies with different porosity, hydration, mechanical stability, and accessibility of binding sites. If a sorbent is later advanced toward optical sensing, the same morphological variables will additionally influence thickness and light scattering.

Recent adsorptions work also shows why pristine and engineered fibroin should not be conflated. Pilley et al. used powdered silk fibroin for Fe removal and reported 98% removal under optimized conditions, a Langmuir maximum capacity of 12.82 mg g^−1^, and reuse over five cycles with only a marginal decrease in efficiency [21]. By contrast, the silk-fibroin-composite literature includes chelating membranes, PEI-containing hydrogels, aerogels, and other chemically modified materials with much larger capacities [14]. Those gains arise from new functional groups and network architectures and therefore cannot be assigned to native fibroin chemistry alone.

### 3.2. Banana and Orange Peels: Heterogeneous Polymer-Rich Matrices

Banana and orange peels differ fundamentally from isolated pectin because they are multicomponent biological materials. Their cell walls contain pectic polysaccharides together with cellulose, hemicellulose, lignin, and minor constituents whose proportions depend on botanical variety, maturity, processing, and the method used to prepare the sorbent. Consequently, the term banana peel biosorbent or orange peel biosorbent does not describe a single chemically uniform polymer.

This heterogeneity is one reason why adsorption capacities reported for fruit-peel materials should not be interpreted solely in terms of one functional group. Hydroxyl groups occur throughout cellulose, hemicellulose, and pectic polysaccharides, whereas carboxyl groups are particularly associated with galacturonic-acid-rich pectic domains. Phenolic and other oxygen-containing sites may also be contributed by lignin and minor components. The surface charge therefore represents the combined behavior of several polymer fractions.

The early comparative study of Annadurai et al. demonstrated adsorption of Cu(II), Co(II), Ni(II), Zn(II), and Pb(II) by banana and orange peels [2]. Under comparable conditions, adsorption followed the order Pb(II) > Ni(II) > Zn(II) > Cu(II) > Co(II) for both materials, and adsorption increased as the pH was raised. The authors also observed corresponding changes in zeta potential. These findings are consistent with an increasing contribution of negatively charged surface sites as acidic groups become less protonated, although they do not establish a unique molecular binding mechanism.

The same distinction should be retained when pectin chemistry is used to interpret fruit-peel adsorption. Banana peel is a confirmed source of extractable pectin. Khamsucharit et al. isolated pectins from five banana varieties using citric acid and reported extraction yields of 15.89–24.08%. The isolated pectins had degrees of esterification between 63.15 and 72.03%, placing them in the high-methoxyl category [4]. These data demonstrate that banana peel contains a substantial pectic fraction and that its chemical characteristics vary with botanical source. They should not, however, be interpreted to mean that the intact banana-peel surface has the same degree of esterification or metal-binding behavior as the subsequently extracted pectin. Extraction changes the chemical environment by separating the polysaccharide from the surrounding cellulose–hemicellulose–lignin matrix and may alter molecular weight, esterification pattern, and accessibility of carboxyl groups.

A comparable consideration applies to citrus peel. Schiewer and Patil examined pectin-rich fruit wastes for Cd(II) sorption and identified citrus peels as particularly promising because they combined relatively high uptake with greater physical stability than some other fruit residues [3]. Equilibrium was reached within approximately 30–80 min depending on particle size in the range investigated. Smaller particles showed faster uptake, while particle size had much less influence on equilibrium capacity [3]. This observation is instructive from a polymer-materials perspective: it indicates that reduction in particle size primarily shortened diffusion or mass-transfer pathways rather than creating a proportionally larger number of equilibrium binding sites.

Such results are directly relevant to biosorbent processing. Particle size, porosity, swelling, and surface heterogeneity can influence both the time required to reach sorption equilibrium and the reproducibility of material handling. If fruit-peel powders are later used as optical phases, these same variables additionally affect diffuse scattering; thus, particle-size control is first a sorption-design requirement and only secondarily an analytical one.

It is also important to avoid an overly simplified application of the egg-box model to untreated fruit peels. The egg-box concept describes cooperative binding of divalent cations by suitably arranged, non-esterified galacturonate sequences in pectin and related polyuronates. In a heterogeneous peel, pectin is only one component of the surface and may have a relatively high degree of methyl esterification. Thus, ion exchange at carboxylate groups is chemically plausible and well supported, but an ordered egg-box-type arrangement should be regarded as a possible local interaction rather than as the established dominant mechanism of an unmodified peel unless direct structural evidence is available.

The distinction between whole peel and extracted polymer is supported by the broader pectin literature. Pectin is not a single linear molecule but a family of galacturonic-acid-rich domains whose composition, methyl esterification, acetylation, and side-chain content vary with source and processing [22,23]. Consequently, assigning a single degree of esterification or a single density of carboxylate sites to an untreated peel is chemically unjustified unless that material has been characterized.

This point is also important for comparisons between banana and citrus sources. The extraction study of Khamsucharit et al. [4] demonstrates that banana peel can yield high-methoxyl pectin, whereas commercial citrus pectins may differ substantially in esterification pattern and acetylation. Recent Polymers coverage of pectin sources and extraction likewise stresses that extraction history changes molecular characteristics and hence material function [13].

### 3.3. Pectin: From Biomass Component to Controllable Anionic Polymer

Isolation of pectin removes much of the compositional uncertainty associated with raw fruit residues and provides a polymer whose chemical and physical property can be varied in a controlled manner. Pectin is a structurally complex family of plant polysaccharides, with homogalacturonan constituting its principal linear domain. Homogalacturonan consists predominantly of α-(1→4)-linked D-galacturonic acid residues, a proportion of whose carboxyl groups are methyl-esterified [24]. Rhamnogalacturonan and other branched regions add structural complexity, but it is the ionization state and distribution of galacturonic-acid carboxyl groups that are particularly important for divalent-metal binding.

The degree of methyl esterification is therefore a central structure–property parameter. Pectins with more than approximately 50% of their galacturonic-acid carboxyl groups methyl-esterified are conventionally classified as high-methoxyl pectins, whereas those below this threshold are classified as low-methoxyl pectins [24]. Lower methyl esterification increases the population of free carboxyl groups capable of ionization and reduces the distance between neighboring negatively charged sites along suitable regions of the chain. However, metal affinity cannot be predicted from the overall degree of esterification alone because the spatial distribution of esterified and non-esterified residues is also important.

The best-established example of controlled ionic organization in pectin is Ca(II)-mediated gelation. In low-methoxyl pectin, sequences of non-esterified galacturonic acid residues can participate in Ca(II)-mediated junction zones commonly described by the egg-box model [24,25]. The model is useful as a representation of cooperative binding between polygalacturonate chains, although modern structural studies indicate that actual pectin–calcium organization is more complex than the earliest idealized scheme. Thus, the egg-box concept should be used as a mechanistic framework rather than as a literal universal structure for every pectin–metal system.

The relevance of this chemistry to toxic-metal sorption was demonstrated directly by Mata et al. [5]. Sugar-beet pectin was extracted, demethylated, and gelled with CaCl_2_ to produce hydrogel and xerogel beads for Cd(II), Pb(II), and Cu(II) uptake. FTIR and SEM characterization supported two principal processes: exchange of the Ca(II) initially incorporated into the gel and interaction of the incoming metal ions with pectin carboxyl groups. The replacement of Ca(II) by sorbed metals was accompanied by structural changes in the gel matrix. This study is particularly valuable because the proposed mechanism was not inferred solely from an adsorption-isotherm fit; it was supported by chemical preparation of the matrix and complementary characterization.

The same group subsequently examined repeated sorption, desorption, and regeneration of sugar-beet pectin xerogels [26]. The beads were used over nine cycles; 0.1 M HNO_3_ was employed for desorption and CaCl_2_ for regeneration. Calcium treatment helped restore binding functionality after acidic elution [26]. These results illustrate an important principle for polymeric sorbents: regeneration is not merely the removal of an analyte but may involve restoration of the ionic state and network organization of the polymer.

Recent work in Polymers further demonstrates the importance of morphology in pectin-based adsorbents. Coin et al. prepared calcium-crosslinked low-methoxyl pectin matrices as solvent-cast films and freeze-dried foams containing immobilized Fe–BTC particles [6]. Although that work was not designed as a toxic-metal sensing study, it showed that changes in matrix morphology and additive dispersion modified porosity, swelling, mass transfer, and adsorption performance. The freeze-dried architecture provided greater accessibility to active regions than the corresponding dense film. This is an important structure–property observation for the present review: identical chemical functionality does not necessarily produce identical adsorption behavior when the three-dimensional organization of the polymer differs.

For biosorbent development, this issue means that crosslink density and morphology must be optimized together rather than independently. A material that swells strongly may provide favorable ion transport but poor dimensional stability, whereas excessive crosslinking can improve mechanical integrity while restricting access to internal binding sites. If direct reflectance sensing is pursued later, the same structural variables will additionally affect scattering and baseline reproducibility. The optimum architecture for adsorption, regeneration, and sensing may therefore not be identical.

Modern descriptions of pectin structure distinguish homogalacturonan, rhamnogalacturonan-I, rhamnogalacturonan-II, and other substituted domains rather than treating pectin as uniform polygalacturonic acid [22,23]. For adsorption, this matters because carboxyl density is concentrated in galacturonic-acid-rich regions, while neutral-sugar side chains and acetylation can alter accessibility, hydration, and chain association. Lara-Espinoza et al. reviewed how these structural features underpin the conversion of pectin into films, gels, and composite materials [27].

The heavy-metal adsorption literature reaches the same conclusion from a different direction. Wang et al. identified degree and distribution of methoxylation, molecular weight, functional groups, ionic strength, pH, and material format as interacting variables in pectin adsorption [11], while Li et al. emphasized that surface carboxyl and hydroxyl groups contribute differently depending on modification and contaminant chemistry [12]. A 2026 review of pectin-derived hydrogels further highlights crosslinking, swelling, mechanical stability, and regeneration as design constraints rather than secondary processing details [28].

### 3.4. Structure–Property Relationships and Modification Strategies Across the Four Platforms

Comparison of the four materials reveals two distinct levels of structural control. Silk fibroin is a semicrystalline protein in which accessibility is governed by the distribution of ordered β-sheet and less ordered hydrated domains. Banana and orange peels are compositionally heterogeneous cell-wall matrices, and their behavior reflects the combined contribution of several natural polymers. Isolated pectin provides the greatest chemical control because the density of ionizable carboxyl groups and the extent of ionic crosslinking can be deliberately altered.

This comparison also explains why literature adsorption capacities obtained for different materials should not be ranked without considering preparation history. Chemical extraction can expose sites that were inaccessible in the intact biomass; demethylation can increase the density of free carboxyl groups; Ca(II) crosslinking can both create a stable network and introduce exchangeable counterions; grinding can shorten diffusion paths; and freeze-drying can generate a highly porous architecture. Each operation changes the polymer material itself and therefore changes the meaning of the measured adsorption capacity.

For the same reason, the terminology used in the remainder of this review should distinguish carefully between raw polymer-rich biomass, isolated biopolymer, and chemically or ionically modified polymer networks. Treating these materials as equivalent “biosorbents” obscures precisely the structure–property relationships that are most relevant to Polymers.

From a biosorbent-design standpoint, four material characteristics emerge as particularly important. The first is the chemical density and ionization state of accessible binding groups. The second is the three-dimensional organization of the polymer, which controls diffusion and mechanical stability. The third is the extent to which processing or modification creates new sites rather than merely exposing native ones. The fourth is regeneration stability. Optical reproducibility becomes an additional criterion only when a sorbent is deliberately advanced toward sensing. Together, these parameters provide the basis for evaluating the sorption data in Section 4.

For a more explicit structure–property–performance analysis, six variables should be interpreted in sequence rather than independently. (1) Accessible functional-group density determines the potential population of binding sites, but only the ionized and sterically accessible fraction contributes under a given pH. (2) Degree and distribution of pectin esterification control the density and spacing of free carboxylates. (3) Ionic or covalent crosslinking changes network stability and swelling while potentially restricting diffusion to internal sites. (4) Porosity and particle or pore architecture regulate mass-transfer length and accessible surface area. (5) Swelling governs ion transport in hydrated polymer networks but can reduce dimensional stability. (6) Chemical modification may expose native sites or create new ligands and therefore must be interpreted against a matched untreated precursor. Performance should consequently be expressed as the combined outcome of binding-site chemistry, transport, morphology, and structural stability, not as adsorption capacity alone.

## 4. Metal-Ion Sorption: Performance, Mechanisms, Controlling Factors, and Metal-Specific Trends

The performance of a polymeric biosorbent is commonly described in terms of equilibrium capacity, removal efficiency, kinetic behavior, selectivity, and regeneration. These parameters are useful for comparing materials only when experimental conditions and material preparation are considered together with the numerical values. Solution pH, metal speciation, initial concentration, sorbent dose, particle size, temperature, ionic composition, and polymer modification can each change the apparent adsorption behavior.

This limitation is particularly important for the selected waste-derived platforms. Raw banana and orange peels are chemically heterogeneous plant matrices; isolated pectin is a more defined polysaccharide; Ca-crosslinked or grafted pectin-rich materials are deliberately reorganized networks; and silk fibroin is a protein-based semicrystalline material. Differences in capacity therefore reflect both metal affinity and material architecture. The precursor, preparation route, and chemical state of the polymer must remain explicit in every comparison. The principal structure–metal binding relationships across the four selected biosorbent matrices are illustrated in Figure 2.

For the same reason, the discussion below distinguishes experimentally measured adsorption capacity, model-derived maximum capacity, removal percentage, and selectivity. These quantities are not interchangeable. A large q_max_ is useful for material screening but does not by itself demonstrate rapid kinetics, preference in mixed-ion systems, regeneration stability, or suitability for a later sensing application.

The standardized, source-traceable adsorption data are summarized in Table 1.

### 4.1. Silk Fibroin: Sorption Behavior and Reusability

The study of Huang et al. provides the principal experimental basis for considering silk fibroin as a metal-binding material in the present review [7]. Fibroin isolated from *Bombyx mori* cocoons was investigated for Pb^2+^, CrO_4_^2−^, Cu^2+^, and Co^2+^. The study included equilibrium, kinetic, thermodynamic, and regeneration experiments rather than relying on removal percentage alone [7].

The source-reported capacities used in this review are 6.01 mg g^−1^ for Pb^2+^, 13.2 mg g^−1^ for CrO_4_^2−^, and 2.29 mg g^−1^ for Cu^2+^ [7]. More important for its possible use as a reusable analytical phase is the demonstrated observation that EDTA treatment regenerated the material and that its sorption capacity remained unchanged over ten sorption–regeneration cycles [7].

Thermodynamic analysis led Huang et al. to characterize metal uptake as spontaneous and endothermic and to classify the interactions as predominantly physical under the conditions investigated [7]. This interpretation should be retained at the level demonstrated by the original study. It would be inappropriate to convert the good fit of a kinetic model into an independent claim of a specific chemical-bonding mechanism.

This is particularly relevant because the modified pseudo-second-order model described the fibroin kinetic data well [7]. Pseudo-second-order behavior is frequently presented in the adsorption literature as evidence for chemisorption; however, contemporary analyses of adsorption kinetics show that pseudo-first- and pseudo-second-order equations can fit systems governed by different physical processes and cannot, by themselves, identify the rate-controlling molecular mechanism [40]. Mechanistic assignments should therefore rely preferentially on spectroscopic evidence, ion-exchange measurements, structural characterization, or thermodynamic observations rather than on kinetic fitting alone.

Chromate also requires separate consideration from the divalent metal cations. CrO_4_^2−^ is an anionic species and cannot be assumed to interact with the fibroin surface through exactly the same electrostatic pathway as Pb^2+^, Cu^2+^, or Co^2+^. Chromium oxidation state, aqueous speciation, and solution pH must therefore be controlled explicitly in comparative sorption studies; the same control would be essential if the material were later evaluated as a sensing phase.

From a biosorbent-design standpoint, the most valuable contribution of the fibroin study is the demonstration that a protein polymer can tolerate repeated metal loading and EDTA-assisted regeneration without loss of bulk sorption performance [7]. This makes regeneration stability a primary selection criterion for future waste-derived fibroin sorbents. If optical sensing is subsequently pursued, retention of surface reflectance and scattering behavior across regeneration cycles would require separate validation.

### 4.2. Banana and Orange Peels: Metal Affinity in Heterogeneous Polymer Matrices

The comparative work of Annadurai et al. remains useful because banana and orange peels were evaluated under the same general experimental framework for Cu^2+^, Co^2+^, Ni^2+^, Zn^2+^, and Pb^2+^ [2]. Under comparable conditions, adsorption decreased in the order Pb^2+^ > Ni^2+^ > Zn^2+^ > Cu^2+^ > Co^2+^ for both materials [2].

For banana peel, the reported maximum uptake values were 7.97 mg g^−1^ for Pb^2+^, 6.88 mg g^−1^ for Ni^2+^, 5.80 mg g^−1^ for Zn^2+^, 4.75 mg g^−1^ for Cu^2+^, and 2.55 mg g^−1^ for Co^2+^ [2]. Corresponding values for orange peel were 7.75, 6.01, 5.25, 3.65, and 1.82 mg g^−1^, respectively [2]. The close similarity of the affinity sequence is notable, but it does not demonstrate that both peels contain identical binding sites. Rather, it suggests that related oxygen-containing polymer fractions in the two plant matrices may provide broadly comparable metal-binding environments under the conditions investigated.

The pH dependence reported in the same study is more informative mechanistically than the absolute capacities. Adsorption increased as pH increased, while changes in zeta potential accompanied the change in metal uptake [2]. These observations are consistent with reduced protonation and increased availability of negatively charged surface groups as the solution becomes less acidic. Because banana and orange peels are heterogeneous mixtures of pectin, cellulose, hemicellulose, lignin, and minor constituents, the observed pH response cannot be attributed to pectin carboxyl groups alone.

A useful example of why values from different studies should not be merged without qualification is provided by Anwar et al. [29]. In dried banana-peel powder, Langmuir analysis gave maximum capacities of 5.71 mg g^−1^ for Cd^2+^ and 2.18 mg g^−1^ for Pb^2+^ [29]. The latter value is considerably lower than the 7.97 mg g^−1^ Pb^2+^ value reported by Annadurai et al. [2]. This difference should not be treated as a contradiction. The studies used different preparation procedures, concentration regimes, sorbent doses, and optimization conditions. It instead illustrates the sensitivity of apparent adsorption capacity to experimental design.

For a critical review, this variability is more informative than selecting the largest published value. It shows that “banana peel” cannot be treated as a chemically invariant material. Botanical origin, drying, grinding, washing, chemical treatment, and storage can alter both the accessible polymer surface and the apparent adsorption capacity. Standardizing these variables is therefore necessary before meaningful comparison or scale-up; if optical sensing is later considered, particle morphology and surface composition will also influence light scattering.

Accordingly, comparisons in this section are quantitative only when materials were tested within the same study under matched conditions. Comparisons between Annadurai et al. [2], Anwar et al. [29], and other independent studies are intentionally qualitative because precursor preparation, concentration range, sorbent dose, pH, and model choice differ. Table 1 now applies the raw biomass/isolated biopolymer/modified material/composite classification consistently so that material transformation remains visible beside every reported performance value.

### 4.3. pH Dependence and the Role of Pectin-Rich Binding Sites

The studies of Schiewer and Patil provide particularly useful evidence for connecting citrus-peel sorption with pectin chemistry [3,30]. In their pH-dependent investigation, orange peels, lemon peels, and a protonated pectin-rich material exhibited Cd^2+^ Langmuir capacities in the range of 0.7–1.2 meq g^−1^, corresponding to approximately 39–67 mg g^−1^ of Cd per gram of dry biosorbent [30]. These values refer to a group of citrus-derived materials and should therefore not be quoted as the capacity of a single isolated pectin preparation.

Potentiometric titration revealed acidic sites with apparent pKa values of approximately 3.8, 6.4, 8.4, and 10.7, with a total site concentration of 1.14 m_eq_ g^−1^ [30]. Modeling showed that the decrease in metal uptake at low pH could be explained by competition between H^+^ and Cd^2+^ for available sites [30]. Importantly, the authors found that a simplified model based on a major acidic site with pKa ≈ 3.8 could reproduce the adsorption behavior without requiring every titratable group to be treated as an independent metal-binding site [30].

This observation is chemically consistent with the role expected for galacturonic-acid carboxyl groups. It also provides stronger mechanistic evidence than a simple correlation between increasing pH and increasing adsorption. Nevertheless, untreated citrus peel should still be distinguished from isolated pectin because cellulose, hemicellulose, lignin, residual soluble constituents, and the heterogeneous distribution of pectin affect accessibility of these sites.

Particle size also influenced the kinetics of Cd^2+^ uptake by pectin-rich fruit wastes. Schiewer and Patil reported equilibrium times of approximately 30–80 min for particle sizes between 0.18 and 0.9 mm, with smaller particles exhibiting faster uptake while particle size had considerably less influence on equilibrium capacity [3]. This result suggests a substantial mass-transfer contribution to the observed rate and shows why particle-size fraction should be standardized when comparing waste-derived biosorbents. Such standardization would also reduce optical-scattering variability if the material is later evaluated by diffuse reflectance.

Matched-material experiments provide stronger mechanistic evidence than isolated maximum capacities. Schiewer and Iqbal removed pectin from orange peel and separately tested the extracted pectic acid; Cd binding decreased after depectination but did not disappear, while pectic acid showed the highest binding capacity [35]. The result demonstrates both the importance of pectin and the contribution of other carboxyl- and hydroxyl-containing peel components.

Selectivity data from purified pectins are also internally variable. Dronnet et al. reported Cu^2+^ ~ Pb^2+^ >> Zn^2+^ > Cd^2+^ ~ Ni^2+^ >= Ca^2+^ for citrus and sugar-beet pectins [41], whereas Kartel et al. found Pb^2+^ >> Cu^2+^ > Co^2+^ > Ni^2+^ >> Zn^2+^ > Cd^2+^ and showed that preferred ions depended on pectin source [42]. Rather than selecting one sequence as universal, these results should be read as evidence that esterification, acetylation, ionic strength, and polymer origin alter apparent affinity.

### 4.4. Isolated Pectin and Ionically Crosslinked Pectin Networks

Evidence obtained with isolated pectin provides a clearer relationship between macromolecular structure and metal uptake. Khotimchenko et al. compared high-esterified pectin, low-esterified pectin, and insoluble calcium pectate for Pb^2+^ adsorption [31]. Maximum lead binding for the pectin preparations occurred at approximately pH 7–8, and the largest amount of Pb^2+^ was bound by the pectin with the lowest degree of esterification [31]. This experiment directly supports the importance of non-esterified galacturonic-acid residues rather than merely inferring their role from the composition of raw fruit residues.

Mata et al. provided additional mechanistic evidence using pectin extracted from sugar-beet pulp, subsequently demethylated and gelled in the presence of CaCl_2_ [5]. Both hydrogel and xerogel forms sorbed Cd^2+^, Pb^2+^, and Cu^2+^. FTIR and SEM analyses, together with changes in calcium content, supported a mechanism involving displacement of Ca^2+^ from the pectin network and interaction of incoming metal ions with carboxyl groups [5]. The reported kinetic uptake followed the order Cu > Pb > Cd, whereas the Langmuir affinity constant followed Pb ≫ Cu > Cd [5]. These two sequences describe different properties—rate-related uptake and equilibrium affinity—and should not be treated as equivalent selectivity rankings.

The Ca^2+^-exchange evidence is especially important because it provides an experimentally supported basis for discussing the egg-box-type organization introduced in Section 3. The mechanism is more defensible for deliberately demethylated, Ca-crosslinked pectin than for raw fruit peel because the polymer preparation and ionic state of the gel were experimentally controlled.

The relevance of polymer architecture is also evident in the pectin-based study of Wang et al. published in Polymers [32]. A pectin microsphere alone exhibited a Langmuir maximum Pb^2+^ capacity of 120.19 mg g^−1^, whereas incorporation of activated carbon progressively increased the capacity, reaching 279.33 mg g^−1^ for the pectin/activated–carbon formulation with a 2:3 ratio [32]. The corresponding Langmuir fit for the latter material gave R^2^ = 0.996 [32]. SEM showed increased porosity after activated–carbon incorporation, and the authors attributed the improved performance to the combined effects of porosity, active surface area, ion exchange, complex formation, and the activated–carbon phase [32].

This comparison is useful because it demonstrates that the adsorption behavior of a pectin-based material is not determined by pectin chemistry alone. The original pectin microsphere and the pectin/activated–carbon composite contain the same principal polysaccharide, yet their maximum Pb^2+^ capacities differ by more than a factor of two [32]. Morphology and composite formulation are therefore design variables rather than secondary characteristics.

For the present review, however, the composite value of 279.33 mg g^−1^ should not be compared directly with the 6–8 mg g^−1^ values reported for unmodified fruit peels. The materials differ in polymer isolation, crosslinking, porosity, presence of activated carbon, accessible surface area, and experimental concentration range. Such a comparison would exaggerate the importance of precursor identity and underestimate the effect of material engineering.

Chemical modification can increase capacity by more than an order of magnitude, but the price of that improvement is loss of comparability with the native biomass. Feng et al. reported Langmuir capacities of 476.1, 293.3, and 162.6 mg g^−1^ for Pb^2+^, Cd^2+^, and Ni^2+^ on graft-copolymer-modified orange peel, corresponding to 4.2-, 4.6-, and 16.5-fold increases over the unmodified material [36]. In a different modification route, NaOH/CaCl_2_-treated orange peel gave 70.73, 209.8, and 56.18 mg g^−1^ for Cu^2+^, Pb^2+^, and Zn^2+^, and Ca^2+^ exchange at pectin carboxyl groups was identified as a major mechanism [37].

The practical relevance of modification should therefore be evaluated against additional criteria: reagent burden, stability, regeneration, selectivity in multi-ion matrices, and reproducibility of material preparation. Lasheen et al. likewise showed that chemically modified orange peel gave higher Cd^2+^, Cu^2+^, and Pb^2+^ uptake than the original peel and explicitly examined desorption, illustrating that a useful sorbent must be assessed as a cycle rather than only at its first equilibrium point [43].

### 4.5. Modified Orange Peel as an Example of Polymer-Matrix Engineering

A recent study by Marzano-Vasquez et al. provides a useful example of how deliberate chemical modification can alter the performance of a fruit-peel matrix [33]. Orange peel was demethoxylated and subsequently crosslinked with Ca^2+^ before Mn^2+^ adsorption. Under the optimized conditions—10 g L^−1^ biosorbent, 100 mg L^−1^ Mn^2+^, pH 5.5 ± 0.1, 25 °C, and 8 h contact time—the material removed 94.8 ± 0.3% of dissolved Mn^2+^ and gave a Langmuir capacity of 29.7 mg g^−1^ [33].

The mechanistic evidence is more important than the isolated capacity value. FTIR confirmed the presence of abundant COO^−^ and OH functionality, while SEM–EDX detected Mn on the loaded material together with a decrease in residual Ca signal, supporting an ion-exchange contribution [33]. The equilibrium data were fitted better by the Freundlich and Sips models (R^2^ = 0.968 and 0.969, respectively) than by the Langmuir model [33]. Consequently, the reported Langmuir capacity of 29.7 mg g^−1^ is useful as a standardized capacity descriptor, but it should not be interpreted as proof that adsorption occurred on an ideal homogeneous monolayer.

This distinction is important for the design philosophy of the present review. Chemical demethoxylation increases the availability of carboxylate sites, whereas Ca^2+^ crosslinking reorganizes the pectin-containing matrix and introduces exchangeable divalent counterions. The resulting sorbent is therefore a chemically engineered polymer-rich material and should not be presented as equivalent to untreated orange peel.

Chromium provides the clearest example of why metal speciation must be incorporated into sorption comparisons. Untreated banana peel showed rapid Cr(VI) uptake with an optimum near pH 2 [38], while natural orange peel in a recent study reached approximately 97.8% removal after 90 min and a reported capacity of 4.96 mg g^−1^ under acidic conditions [39]. Acid activation or acrylonitrile grafting can further increase apparent performance [44,45], but these treatments simultaneously change surface protonation, functional-group density, and transport properties. Cr(VI) values from such materials should therefore be interpreted as modification-specific rather than as an intrinsic property of banana or orange peel.

A parallel lesson emerges from renewable polysaccharides more broadly. Recent work in Polymers shows that selective sorption depends on the identity and density of acidic groups and on competition among ions [46]. This supports the present review’s decision to treat pH, ionic strength, competing ions, and polymer source as primary explanatory variables rather than as experimental details.

### 4.6. What Can—And Cannot—Be Concluded from the Sorption Data

The available evidence supports several conclusions. First, all four material classes have experimentally demonstrated affinity toward environmentally relevant metal species. Second, polymer structure matters: esterification state, ionic crosslinking, porosity, particle size, and composite formulation can alter sorption behavior as strongly as the identity of the biological precursor. Third, pH is a major control variable because it changes both polymer-site protonation and metal speciation.

At the same time, the literature does not support a universal ranking such as “pectin > orange peel > banana peel > fibroin”. Such a ranking would combine experiments performed with different metals, concentrations, material forms, temperatures, and equilibrium models. The numerical values in Table 1 should therefore be interpreted as source-specific benchmarks, not intrinsic constants of the polymers.

Likewise, model fitting must be separated from mechanistic proof. A Langmuir fit does not demonstrate that a heterogeneous biological surface is physically homogeneous, and a pseudo-second-order kinetic fit does not by itself demonstrate chemisorption [40]. Stronger mechanistic evidence is provided when adsorption measurements are accompanied by FTIR, XPS, SEM–EDX, ion-release measurements, changes in zeta potential, controlled esterification, or direct comparison of crosslinked and non-crosslinked matrices.

This distinction is equally important for practical material selection. The biosorbent with the highest capacity is not necessarily the best platform if it is slow, mechanically unstable, difficult to regenerate, or poorly selective in mixed-ion water. Conversely, a material with a more moderate capacity may be preferable if it can be prepared reproducibly from waste, retains structure during reuse, and responds predictably to controlled modification. Optical sensing, where pursued, introduces an additional requirement for reproducible surface morphology and reflectance.

The adsorption literature therefore provides the chemical foundation for selecting precursor–metal combinations and rational modification routes, but it does not support a single universal ranking of the four materials. A more informative next step is a metal-specific synthesis that identifies where evidence is strongest, where selectivity remains uncertain, and which combinations are most suitable for controlled experimental development. That synthesis is provided in Section 4.7.

### 4.7. Metal-Specific Critical Assessment and Experimental Priorities

The usefulness of the four polymer-based matrices cannot be assessed independently of metal identity. The ions considered here differ in charge, aqueous speciation, coordination chemistry, competition for oxygen- and nitrogen-containing donor groups, and response to pH. A material that performs well for Pb(II) cannot therefore be assumed to show equivalent behavior toward Cr(VI), Cd(II), or Mn(II). Metal-specific interpretation is needed before selecting a precursor and modification route for experimental development.

The comparison below integrates adsorption magnitude, strength of mechanistic evidence, matrix dependence, regeneration where available, and—only as a secondary criterion—the existence of a plausible optical response. This arrangement prevents analytical prospects from dominating material selection while preserving them as a possible later function.

The metal-specific evidence and its implications for biosorbent design are summarized in Table 2.

#### 4.7.1. Lead(II): Strong Sorption Evidence and a Priority Pectin-Binding Target

Among the divalent metals considered, Pb^2+^ has the most consistent evidence of strong interaction with pectin-rich materials. Annadurai et al. reported Pb^2+^ uptake of up to 7.97 mg g^−1^ for banana peel and 7.75 mg g^−1^ for orange peel under directly comparable conditions [2]. The value of that study lies less in the magnitude of the capacities than in the matched experimental design, which makes the peel-to-peel comparison defensible.

The structural role of pectin is clearer in purified materials. Khotimchenko et al. showed that Pb^2+^ binding was greatest for the pectin with the lowest degree of esterification [31]. Dronnet et al. placed Pb^2+^ together with Cu^2+^ at the top of the selectivity series for citrus and sugar-beet pectins [41], whereas Kartel et al. ranked Pb^2+^ distinctly above Cu^2+^, Co^2+^, Ni^2+^, Zn^2+^, and Cd^2+^ [42]. The two studies agree on strong Pb affinity but disagree on the remainder of the sequence, indicating that pectin origin and substitution pattern must be retained in any mechanistic interpretation.

From a biosorbent perspective, Pb^2+^ is therefore one of the best-supported target ions for pectin-rich materials. Its optical behavior is a secondary issue: the 2 2 μg L^−1^ detection limit obtained with xylenol-orange-modified silica demonstrates that solid-phase reflectance can be sensitive when a chromophore is deliberately introduced [47], but strong Pb adsorption should not be equated with an intrinsic visible response of the natural polymer.

#### 4.7.2. Cadmium(II): Strong Evidence for the Role of Pectin, but No Universal Selectivity Pattern

Cd^2+^ illustrates the difference between capacity and selectivity. Banana peel removes Cd rapidly [34], and Anwar et al. reported a Langmuir q_m_ of 5.71 mg g^−1^ [29]. Yet the more informative experiment is the orange-peel deconstruction study: pectic acid showed the highest Cd binding, depectinated peel the lowest, and native/protonated peels intermediate values [35]. Pectin is therefore important but not the only contributing polymer fraction.

Purified-pectin selectivity data caution against calling Cd a preferred pectin ion. Dronnet et al. placed Cd below Zn and far below Cu/Pb [41], while Kartel et al. placed Cd at the bottom of their sequence [42]. A single-metal adsorption capacity can thus be substantial even when the same ion is weakly competitive in a multimetal environment. This is likely to become important in natural waters containing Ca^2+^, Mg^2+^, Cu^2+^, Pb^2+^, and other competing cations.

For material development, Cd^2+^ is especially valuable as a mechanistic probe because matched depectination experiments already distinguish the contribution of pectin from that of the remaining peel matrix. A future optical application would be more difficult because Cd^2+^ itself has weak intrinsic visible absorption; any useful signal would probably have to originate from a polymer structural or chromophoric change rather than from the ion alone.

#### 4.7.3. Chromium: Speciation Must Precede Any Discussion of Affinity

Chromium cannot be treated as a single generic metal analyte. Cr(VI) in aqueous solution is present as oxoanionic species whose distribution depends on pH, whereas Cr(III) is cationic and has different coordination chemistry. The acidic optimum observed for untreated banana peel (approximately pH 2) [38] and for natural orange peel [39] is therefore not contradictory to the higher-pH behavior observed for divalent cations on carboxylate-rich pectin surfaces; it reflects a different electrostatic and speciation regime.

Material modification can further alter Cr(VI) behavior. Acid-activated banana peel [44] and acrylonitrile-grafted banana peel [45] showed enhanced removal under acidic conditions, but these treatments change surface chemistry and cannot be used as evidence for the intrinsic behavior of native peel. Comparisons should therefore separate untreated, activated, and grafted materials explicitly.

The Cr(III)-Eriochrome Black T-fibroin method provides a useful downstream benchmark [48], but the primary material-design issue remains chromium speciation. Cr(VI) and Cr(III) should be treated as distinct sorption targets, with pH and oxidation state explicitly controlled before any comparison of capacities or modification strategies is attempted.

#### 4.7.4. Copper(II): Strong Pectin Affinity and a Promising Functional Target

Cu^2+^ is particularly attractive because both pectin affinity and optical chemistry are favorable. Dronnet et al. placed Cu^2+^ approximately equal to Pb^2+^ at the top of their pectin selectivity series [41], and Kartel et al. ranked it immediately below Pb^2+^ [42]. Ca-pectin gels also provide mechanistic evidence for Cu uptake through carboxyl-group interaction and Ca^2+^ displacement [5].

Raw-peel capacities are lower than those of engineered or isolated pectin systems, which is not surprising given dilution of accessible pectic domains by cellulose, hemicellulose, lignin, and other components. The NaOH/CaCl_2_-modified orange-peel study, for example, produced a much higher Cu^2+^ Langmuir capacity than the untreated-peel benchmark and linked uptake to Ca^2+^ exchange [37]. This supports structure–property control but does not justify back-assigning the modified capacity to native peel.

Cu^2+^ is consequently one of the strongest candidates for comparative biosorbent experiments because pectin affinity, Ca^2+^ exchange, and modification effects are all well represented in the literature. The PAN/filter-paper reflectance method [49] shows that Cu can also provide an optical signal when selective ligand chemistry is supplied, but this should be regarded as an optional functional extension after sorption selectivity and regeneration have been established.

#### 4.7.5. Nickel, Cobalt, and Zinc: Sorption Is Established, but Selectivity Remains Matrix-Dependent

The evidence for Ni^2+^, Co^2+^, and Zn^2+^ is less internally consistent. Untreated banana and orange peels showed the common sequence Pb^2+^ > Ni^2+^ > Zn^2+^ > Cu^2+^ > Co^2+^ in the Annadurai study [2], whereas isolated pectins produce different relative orders [41,42]. Kartel et al. further showed that the preferred ion depended on pectin source, with citrus pectin favoring Ni^2+^ more than the other pectins they examined [42].

These discrepancies are scientifically useful. They show that there is no defensible single ‘pectin selectivity sequence’ independent of source, ionic strength, and substitution pattern. They also suggest that Ni^2+^, Co^2+^, and Zn^2+^ may initially be most informative as competing ions in selectivity experiments rather than as the first target metals for optimization of a new waste-derived biosorbent.

Nickel nevertheless provides a useful selectivity benchmark. The Ni-dimethylglyoxime colorimetric solid-phase extraction method demonstrates rapid reflectance quantification when a highly selective ligand is present [50], but for the waste-derived biosorbents themselves, Ni^2+^, Co^2+^, and Zn^2+^ are more informative initially as competing-ion probes for evaluating the robustness of Pb-, Cu-, or Cd-focused materials.

#### 4.7.6. Manganese(II): High Uptake After Matrix Engineering Requires Broader Validation

Mn^2+^ is represented most clearly by the Ca-crosslinked, demethoxylated orange-peel system, which gave 94.8 ± 0.3% removal and a Langmuir capacity of 29.7 mg g^−1^ under optimized conditions using an initial Mn^2+^ concentration of 100 mg L^−1^ [33]. The result demonstrates effective matrix engineering but should not be read as evidence of trace-level analytical performance.

A recent sorption-spectroscopic Mn^2+^ method on a modified polyacrylonitrile carrier provides a useful analytical benchmark [51], but it should not be conflated with the orange-peel system. For the latter, the immediate questions are whether the Ca-crosslinked matrix remains selective in mixed-ion water, whether its long equilibration time can be reduced, and whether regeneration preserves the engineered pectin-rich network.

For orange-peel-derived materials, the next informative experiment is therefore a controlled comparison of untreated and Ca-crosslinked matrices across environmentally relevant concentration ranges, including mixed-ion competition, kinetic behavior, and regeneration. Reflectance screening should follow only after the sorption material has been standardized and its repeatability established.

#### 4.7.7. Priority Ranking for Experimental Development of Waste-Derived Biosorbents

The literature does not justify selecting experimental targets solely by the highest reported q_max_. A more useful ranking for waste-derived biosorbent development considers: strength of sorption evidence; mechanistic support; availability and reproducibility of the precursor; magnitude of improvement produced by modification; selectivity in the presence of competing ions; regeneration stability; and only then the potential for an additional sensing function.

Pb^2+^ and Cu^2+^ should be considered first-line targets for comparative material development. Pb^2+^ has the most consistent evidence of strong binding to pectin-rich matrices, while Cu^2+^ combines high pectin affinity with well-documented Ca^2+^ exchange in engineered gels. Cd^2+^ is a particularly useful mechanistic target because depectination studies already isolate the contribution of the pectic fraction. Chromium should be investigated separately by oxidation state, and Mn^2+^ is valuable for testing the consequences of deliberate Ca-crosslinking of orange-peel matrices.

Ni^2+^, Co^2+^, and Zn^2+^ should initially be included as competing ions rather than prioritized solely as single-component adsorption targets. Their variable positions in published selectivity sequences make them useful for testing whether a newly prepared biosorbent retains performance in multicomponent solutions. This experimental hierarchy is more informative for material development than repeating isolated single-metal q_max_ measurements.

The most informative next-stage experiment is therefore a controlled comparison in which precursor origin, pretreatment, particle-size fraction or film geometry, pH, metal loading, competing ions, and regeneration are evaluated under the same protocol. Raw and modified forms should be tested in parallel so that gains can be attributed to specific structural changes. Only after a reproducible biosorbent has been identified should full diffuse-reflectance spectra be used to test whether the same material can also function as a sensing phase.

## 5. From Biosorbents to Functional Analytical Materials: Prospects for Solid-Phase Optical Sensing

The adsorption studies discussed in Section 4 establish that silk fibroin, fruit-peel matrices, and pectin-based materials can accumulate metal ions from aqueous media. Once a biosorbent has been structurally characterized, shown to be reproducible, and evaluated for regeneration, an additional question can be asked: can metal loading be converted into a measurable response without eluting the analyte back into solution? This section treats diffuse-reflectance sensing as a possible value-added function of a successful biosorbent rather than as the primary basis for selecting the waste precursor.

Diffuse reflectance spectroscopy is well suited to opaque and strongly scattering materials for which conventional transmission measurements are impractical. Nevertheless, quantitative application to heterogeneous biosorbents requires greater experimental control than qualitative comparison of spectra. The measured reflectance is determined not only by absorption associated with the analyte or polymer but also by scattering, particle size, surface roughness, packing density, sample thickness, refractive-index contrast, and hydration state. These factors are especially relevant to natural polymer matrices because their physical organization may change during sorption, washing, drying, and regeneration.

### 5.1. Kubelka–Munk Treatment of Diffuse Reflectance

The two-flux theory developed by Kubelka and Munk provides the most widely used framework for treating diffuse reflectance from strongly scattering media [52,53]. For an optically thick layer, the Kubelka–Munk function is commonly expressed as:

$$F\left(R\infty \right)=\frac{{\left(1\;-\;R\infty \right)}^{2}}{\left(2R\infty \right)}\;=\frac{K}{S}$$

where *R∞* is the diffuse reflectance of a layer of effectively infinite optical thickness, *K* is the absorption coefficient, and *S* is the scattering coefficient [52,53].

The distinction between *R* and *R∞* is important. The simplified expression is appropriate when the sample layer is sufficiently thick for the measured reflectance to become independent of further increases in thickness. If this condition is not satisfied, the measured signal may also depend on the backing and on the physical thickness of the layer [52,54].

When the scattering coefficient remains approximately constant within a series of samples, variation in *F(R∞)* can be related to variation in absorption and, under suitable conditions, to the concentration of the absorbing species. This is the basis for the quantitative use of the Kubelka–Munk transformation. It should not, however, be treated as a universal equivalent of the Beer–Lambert law for solid materials. Christy et al. demonstrated experimentally that particle size affects diffuse-reflectance behavior and may require modification of the conventional treatment for quantitative analysis [55].

This limitation is particularly relevant to the materials considered in this review. Metal uptake can modify the local refractive index, hydration, swelling, surface chemistry, and packing characteristics of a polymeric sorbent. Thus, changes in *F(R∞)* following metal loading cannot automatically be attributed entirely to increased optical absorption. A valid analytical calibration must demonstrate that the scattering contribution is sufficiently stable, or that the applied calibration procedure compensates for its variation.

### 5.2. Sample Morphology as a Source of Analytical Variability

Quantitative diffuse-reflectance measurements are highly sensitive to physical sample preparation. Liang et al. investigated macroporous polymer particles and showed that particle-size distribution, packing density, surface condition, and repacking of the sample could introduce appreciable spectral variation [56]. Their proposed pretreatment procedure combined reproducible packing, normalization, baseline correction, and subsequent Kubelka–Munk transformation. After such treatment, relative deviations between replicate diffuse-reflectance spectra were reduced to below 5% [56].

This observation has direct consequences for banana- and orange-peel powders. In conventional batch sorption experiments, modest variation in particle packing after filtration may have little influence on the calculated aqueous-phase concentration. In a reflectance-based method, the same variation can directly change the analytical signal. Particle-size fraction must therefore be specified rather than described simply as “ground peel”.

The same consideration applies to silk fibroin. A fibrous sample has a directional morphology that differs markedly from a randomly packed powder. Orientation of fibers relative to the incident beam, surface density, thickness of the fiber layer, and residual moisture may influence the measured reflectance. Consequently, calibration should be performed using the same physical geometry used for unknown samples.

Pectin presents a different challenge. It may be measured as a powder, film, hydrogel, xerogel, foam, or microsphere. Each morphology has a different scattering behavior. A calibration established for powdered pectin cannot therefore be transferred automatically to a Ca-crosslinked film or porous foam even when the chemical composition is similar. For polymer-based optical sensing, morphological standardization becomes part of analytical validation rather than merely a materials-characterization issue.

The need to control particle size is supported beyond the classical polystyrene studies. Dai et al. showed a quantitative near-infrared diffuse-reflectance application in which calibration quality changed substantially with particle-size distribution and that a defined particle-size window improved prediction [57]. Although the analyte and matrix differ from the biosorbents reviewed here, the optical lesson is directly transferable: particle-size standardization is part of calibration design, not merely sample preparation.

Colorimetric solid-phase extraction also provides a mature analytical benchmark. Fritz et al. combined solid-phase extraction with handheld diffuse-reflectance measurement for several ions and demonstrated that preconcentration and optical measurement can be integrated on a membrane in approximately one minute [58]. The method depended on immobilized or released complexing reagents, however, and therefore represents an intermediate architecture between conventional solution colorimetry and reagent-free polymer sensing.

### 5.3. Evidence That Polymeric Solids Can Be Quantified by Diffuse Reflectance

Although direct metal determination on the four unmodified matrices remains poorly developed, quantitative diffuse-reflectance measurements on polymeric materials are well established in other applications.

Juang and Storey used diffuse-reflectance FTIR spectroscopy combined with Kubelka–Munk treatment for quantitative determination of the extent of neutralization of Carbopol 974P NF, a crosslinked poly(acrylic acid) polymer [59]. The method distinguished the carbonyl band of unionized carboxylic acid at 1695 cm^−1^ from the carboxylate-related band at 1570 cm^−1^ [59]. A calibration based on derivative spectral ratios was linear over the investigated range from 0 to 100% neutralization [59]. This study is relevant because it demonstrates that changes in the ionization state of carboxyl groups within a crosslinked polymer can be quantified directly from the solid phase.

Pectin provides an even closer precedent. Gnanasambandam and Proctor developed a DRIFTS method for determining the degree of esterification of commercial pectins [9]. The ester carbonyl band, centered at approximately 1756 cm^−1^, showed the highest correlation with titrimetrically determined degree of esterification, with R^2^ = 0.822 for the relationship based on ester-band area [9]. The authors also noted that spectral variation associated with the source of the pectin had to be considered when developing prediction equations [9].

Monsoor et al. subsequently improved this approach by using the ratio of the esterified-carboxyl signal to the total carboxyl signal [10]. The resulting calibration between DRIFTS response and independently determined degree of esterification gave R^2^ = 0.97 [10]. Together, these studies establish two points of particular relevance here. First, pectin can be analyzed quantitatively without dissolution of the polymer. Second, the optical calibration depends on both polymer chemistry and sample provenance, which argues against the use of a universal calibration for chemically heterogeneous.

Representative source-traceable examples relevant to quantitative diffuse-reflectance analysis are summarized in Table 3.

### 5.4. Immobilized Reagents and Direct Polymer Response Are Different Analytical Concepts

Solid-phase spectrophotometry based on immobilized chromogenic reagents provides an important benchmark for the present work. Zaporozhets and Tsyukalo immobilized xylenol orange on silica and used the resulting solid reagent for Pb^2+^ preconcentration followed by diffuse-reflectance detection [47]. The reported detection limit was 2 µg L^−1^, and the method was applied to natural water and food samples [47]. This work demonstrates the analytical power of combining sorption and color development in a single solid phase.

An even more relevant example was reported in 2026 by Smanova et al. for Cr(III) determination using Eriochrome Black T immobilized on silk fibroin [48]. Reflectance spectra were recorded directly from the fibrous phase and evaluated in Kubelka–Munk coordinates [48]. The optimized system operated at pH 8 with a spectral response centered at 618 nm and provided a detection limit of 1.2 µg L^−1^ [48]. The reported calibration was linear for 5–50 µg of Cr(III), with R^2^ = 0.9991, and the analysis time was 10 min [48]. Application to industrial wastewater produced relative errors not exceeding 6% [48].

The importance of this result for the present review is twofold. First, it establishes experimentally that silk fibroin can function as a support for reflectance-based metal determination rather than only as a removal sorbent. Second, it requires a more precise definition of the remaining research gap.

The Fib–EBT–Cr(III) system is not a reagent-free optical response of pristine fibroin. The chromophore responsible for analytical sensitivity is an immobilized azo reagent, Eriochrome Black T, whose interaction with Cr(III) creates the measured spectral change [48]. The polymer functions simultaneously as a carrier and preconcentration phase, while molecular recognition and color generation are substantially provided by the immobilized reagent.

This design is analytically effective but conceptually different from a system in which the natural polymer or unmodified biomass itself develops a measurable optical response after metal uptake. The latter route would eliminate not only elution but also the separate chromogenic reagent. Whether this more demanding form of direct sensing is feasible remains to be established for the matrices reviewed here. The distinction between the conventional sorption–elution workflow and direct solid-phase optical readout is illustrated in Figure 3.

Selectivity is likely to be at least as important as sensitivity. In the PAN/filter-paper Cu^2+^ method, Fe^3+^, Ni^2+^, Co^2+^, Cd^2+^, Mn^2+^, Zn^2+^, and Pb^2+^ produced significant interference until masking agents were introduced [49]. The Ni-DMG method achieved selectivity by exploiting a well-established ligand reaction rather than the surface chemistry of the support [50]. These examples indicate that a reagent-free biosorbent sensor must demonstrate an intrinsic selectivity mechanism or accept that multivariate spectral analysis or selective modification may be necessary.

Recent polymer-supported Mn^2+^ work reinforces the same point. Nitroso-R-salt immobilized on a modified polyacrylonitrile fiber produced a 0.003 mg L^−1^ detection limit and good agreement with ICP-MS in soil samples [51], but Fe(III), Ni(II), and Co(II) remained important interferents. Thus, transferring a successful adsorption material into analysis requires a second layer of evidence: the optical response must survive realistic competition and matrix effects.

### 5.5. Requirements for Reagent-Free Direct Optical Detection

For an unmodified biosorbent to function as a direct optical sensing phase, metal binding must cause a reproducible change in the absorption characteristics of the solid that can be distinguished from changes caused by hydration, morphology, or scattering. This requirement is not automatically satisfied by successful adsorption.

Several situations are possible. A transition-metal ion may form a surface complex with sufficient visible absorption to alter the color of the loaded polymer. Alternatively, metal binding may modify existing chromophoric groups within lignin- or protein-containing matrices. For ions such as Cd^2+^ and Pb^2+^, which generally do not provide intense intrinsic visible absorption under ordinary coordination conditions, a direct color response may be weak. In such cases, reagent immobilization or another signal-amplification strategy may ultimately prove necessary.

The analytical development of any proposed system should therefore begin with full reflectance spectra of the unloaded and metal-loaded material rather than immediately constructing a single-wavelength calibration. Spectral changes should be examined as a function of metal loading, pH, ionic strength, and competing ions. Only after identifying a reproducible spectral region should a quantitative calibration be established.

For powders, the physical variables that should be controlled include particle-size fraction, sorbent mass, thickness of the measured layer, packing density, drying or hydration protocol, orientation and geometry of the sample cup, and the reference material and instrumental geometry. For fibrous or film-like polymers, thickness, orientation, surface roughness, and hydration history should be controlled to the same extent.

This requirement follows directly from the optical theory. The simplified Kubelka–Munk relationship assumes conditions under which scattering can be treated in a sufficiently stable manner. Finite thickness and deviations from ideal diffuse scattering can introduce systematic errors [52,54]. Consequently, linearity of *F(R∞)* versus analyte concentration should be demonstrated experimentally rather than assumed from the theoretical expression.

### 5.6. Evidence Levels for Translating a Biosorbent into a Sensing Material

A useful distinction can be made between four successive levels of evidence:

**Level I—sorption:** the polymer removes the analyte from solution.

**Level II—spectral response:** metal loading produces a reproducible change in the reflectance spectrum.

**Level III—quantitative calibration:** the optical response is related to analyte amount or concentration over a defined working range.

**Level IV—analytical validation:** the method has demonstrated limits of detection and quantification, precision, selectivity, recovery, robustness, and performance in real samples.

The studies summarized in Section 4 largely establish Level I for the four biosorbent platforms. The pectin DRIFTS studies establish that quantitative solid-state spectroscopic calibration is possible for a structural property of pectin [9,10], whereas the immobilized-reagent systems demonstrate quantitative metal determination on designed solid supports [47,48,49,50,51,58]. These are important precedents, but they do not convert adsorption capacity into analytical sensitivity.

By contrast, within the literature examined for this review, evidence remains insufficient to establish Levels III and IV for reagent-free determination of metal ions from the intrinsic diffuse-reflectance response of unmodified silk fibroin, banana peel, orange peel, or isolated pectin. This wording is deliberately narrower than stating that diffuse-reflectance metal sensing has never been performed with these materials, because the recently reported Fib–EBT system clearly demonstrates otherwise [48].

This distinction defines a more defensible research question. The relevant task is no longer to demonstrate that a natural polymer can be used as a carrier for a conventional chromogenic reagent. That principle is already supported experimentally. The more fundamental polymer-science question is whether chemical and structural changes produced by metal binding to the polymer itself can generate a sufficiently stable optical response for quantitative analysis.

For any material advanced to Levels III–IV, analytical validation should include the stated procedure used to calculate LOD and LOQ, calibration range and model, within-run precision, independently prepared-batch precision, matrix-spike recovery, interferent tolerance, blank and baseline drift, and retention of the optical response after regeneration. Batch-to-batch reproducibility should be evaluated with independently prepared sorbent batches rather than repeated readings from a single batch. Sorption selectivity should not be used as a surrogate for analytical selectivity because optical matrix effects and scattering variability can remain even when chemical uptake is preferential.

### 5.7. Implications for Polymer Design

The discussion above also indicates that development of an optical sensing phase should not be separated from polymer engineering. Binding-site density affects analyte uptake, but morphology controls both mass transport and scattering. Crosslinking may improve dimensional stability while reducing swelling and limiting access to internal sites. A highly porous structure may accelerate sorption but produce larger variation in diffuse scattering. Conversely, a dense film may provide excellent optical reproducibility while giving slow equilibration.

The optimal material for direct reflectance sensing may therefore not be the material with the highest equilibrium adsorption capacity. A lower-capacity polymer with reproducible thickness, controlled surface chemistry, and stable reflectance may provide better analytical performance than a highly porous powder with heterogeneous scattering.

For pectin, this consideration favors systematic comparison of powders, Ca-crosslinked films, xerogels, and porous structures rather than selection on q_max_ alone. For fibroin, fibrous mats or standardized films may offer greater optical reproducibility than randomly packed fragments. For banana and orange peels, fractionation by particle size and control of drying conditions will be essential before meaningful calibration can be attempted.

Thus, transition from biosorption to sensing should be regarded as a second-stage materials-development problem. The first-stage objective remains a reproducible and regenerable biosorbent. Only then should optical geometry, scattering stability, calibration, selectivity, and real-sample validation be optimized for an optional sensing function.

## 6. Regeneration, Sustainability, and Practical Applicability

The practical value of a waste-derived biosorbent is determined not only by first-cycle adsorption capacity but also by the amount of processing required to prepare the material, its stability during reuse, the chemistry of desorption, and the fate of the recovered metal. This perspective is particularly important for banana and orange peels, fibroin residues, and extracted pectin because a renewable precursor can lose part of its environmental advantage if extensive chemical modification, repeated high-temperature drying, or reagent-intensive regeneration is required. Green assessment should therefore be applied to the complete material-use cycle rather than to precursor origin alone [8,60].

### 6.1. Regeneration and Reuse as Material-Performance Criteria

The studies reviewed above demonstrate that regeneration is feasible for several of the selected platforms, but the evidence is uneven. Fibroin retained its bulk sorption capacity over ten EDTA-assisted cycles in the study of Huang et al. [7]. Demethylated Ca-crosslinked pectin xerogels were desorbed with 0.1 M HNO_3_ and subsequently regenerated with CaCl_2_ over repeated cycles [26]. Modified orange-peel systems have likewise been evaluated for desorption and reuse [33,43]. These results are encouraging, but they also show that regeneration is a chemical operation with its own reagent demand and waste stream. The proposed staged pathway from biosorbent development to optional solid-phase sensing is shown in Figure 4.

Desorption and regeneration should not be treated as synonymous. Desorption measures removal of the retained metal, whereas regeneration additionally requires restoration of the sorbent’s binding functionality and physical integrity. Recent critical analysis of spent adsorbents emphasizes that eluent selection, metal recovery, reuse efficiency, secondary waste generation, and safe disposal are interdependent sustainability variables [61]. For polymeric biosorbents, repeated-cycle assessment should therefore report capacity retention together with mass loss, dimensional or particle stability, and—where possible—changes in FTIR, morphology, or surface chemistry.

Comparative regeneration and reuse evidence for the selected natural-polymer biosorbents are summarized in Table 4.

The comparison also exposes a reporting gap: desorption efficiency, capacity retention, mass recovery, chemical/structural stability, and metal recovery are rarely reported together. Future regeneration claims should therefore separate these endpoints and provide adsorption–desorption mass balances rather than using successful elution alone as evidence of reusability.

### 6.2. Processing Burden of a Waste-Derived Biosorbent

Minimal processing favors low cost and low reagent consumption, but raw biomass is chemically heterogeneous and may provide poor batch-to-batch reproducibility. Washing, drying, milling, and sieving are already necessary to standardize fruit-peel powders. Extraction of pectin, demethylation, Ca^2+^ crosslinking, grafting, or fibroin regeneration can improve binding-site accessibility and mechanical stability, but each additional step consumes chemicals, water, and energy. ComplexGAPI was developed specifically to include processes that precede the analytical procedure, such as preparation of functional materials [62], while the principles of Green Sample Preparation emphasize renewable or reusable materials together with reduced solvent/reagent consumption, waste, energy, and processing time [63]. These concepts are directly transferable to biosorbent preparation even when the final application is water treatment rather than analysis.

The central design trade-off is therefore not ‘raw waste versus modified material’ in absolute terms. The relevant question is whether a processing step produces a sufficiently large and reproducible gain in capacity, selectivity, kinetics, mechanical stability, or reuse to justify its additional burden. For example, demethylation and Ca^2+^ crosslinking can create a more controllable pectin-rich network, whereas aggressive grafting may yield very high capacities but moves the material farther from the chemistry and low-processing advantage of the original waste precursor.

### 6.3. Green and Practical Assessment

Formal greenness scores should be calculated only after a complete experimental protocol has been defined. AGREE evaluates a full analytical procedure against the twelve principles of green analytical chemistry [8], GAPI maps environmental impact from sampling to final determination [64], and AGREEprep focuses on the sample-preparation stage [65]. These tools can be useful if a biosorbent is ultimately developed into an analytical phase, but assigning a numerical score before sample volume, sorbent mass, reagent volumes, drying conditions, energy use, regeneration, and waste generation are experimentally known would create an artificial comparison.

Practicality must be considered together with environmental performance. White Analytical Chemistry explicitly integrates analytical quality, ecological attributes, and practical utility [66], while BAGI provides a complementary framework for method applicability [67]. The same principle applies to biosorbents even outside analytical use: a low-reagent material with very slow equilibration, poor selectivity, weak mechanical integrity, or rapid capacity loss may be less useful than a moderately modified material that can be reused reliably. For future sensing applications, the final comparison should also include detection limit, precision, matrix effects, throughput, and instrumentation requirements.

The principal practical and sustainability criteria for biosorbent development are summarized in Table 5.

The most defensible sustainability claim at the present stage is therefore conditional: waste-derived polymer biosorbents have clear potential to reduce reliance on virgin synthetic sorbents and to support material reuse, but the magnitude of this advantage depends on processing intensity, regeneration chemistry, selectivity, and end-of-life management. These parameters should be measured alongside adsorption performance in future comparative experiments rather than added retrospectively after the highest-capacity formulation has been selected.

## 7. Research Gaps and Design Guidelines for Waste-Derived Biosorbents

The literature reviewed in the preceding sections confirms that silk fibroin, banana peel, orange peel, and pectin-containing materials can bind environmentally relevant metal ions. At the same time, the available evidence is considerably stronger for laboratory-scale adsorption than for reproducible material manufacture, competitive sorption, continuous operation, long-term regeneration, or practical deployment. The principal research gap is therefore no longer whether these natural polymer matrices are capable of binding metals. The more relevant question is how to transform variable biological residues into chemically defined, reproducible, regenerable, and application-oriented biosorbent materials. The proposed circular development pathway for a waste-derived biosorbent is illustrated in Figure 5.

This distinction is important because a waste-derived biosorbent occupies an intermediate position between an untreated biological residue and a deliberately engineered polymeric material. Minimal processing preserves low cost and reduces chemical consumption, but it also preserves variability in composition and morphology. More extensive modification can increase binding-site density, selectivity, mechanical stability, or porosity, but may simultaneously increase reagent demand and weaken the environmental and economic arguments for using a waste-derived precursor. The most useful research strategy should therefore seek the minimum level of modification required to obtain reproducible performance, rather than the highest adsorption capacity achievable after unrestricted chemical treatment.

Recent reviews of agro-waste biosorption reach a similar conclusion. Large numbers of materials perform well in synthetic single-metal solutions, whereas considerably fewer studies address real wastewater, competitive adsorption, long-term regeneration, continuous systems, or safe management of exhausted sorbents [68,69,70,71,72]. A recent Polymers review focused specifically on continuous adsorption using lignocellulosic residues emphasized that scaling laboratory modification routes and transferring monocomponent results to multicomponent continuous systems remain major challenges [68]. These limitations provide a useful framework for defining the next stage of research on the four matrices considered here.

### 7.1. Precursor Variability Must Become an Experimental Variable

One of the most important but frequently underestimated problems is the reproducibility of the starting material. Banana and orange peels are not standardized polymers. Their composition can vary with botanical variety, maturity, growing conditions, storage, food-processing history, and the procedure used for washing and drying. The banana-pectin study discussed earlier, for example, showed measurable differences in pectin yield and degree of esterification among different banana varieties [4]. Broader pectin literature similarly demonstrates that source and extraction history affect molecular weight, esterification, acetylation, branching, and downstream physicochemical behavior [13,22,23].

Consequently, future work should not describe a material simply as “banana peel powder” or “orange peel biosorbent”. At minimum, the biological source, state of the residue, washing procedure, drying temperature, drying time, grinding procedure, particle-size fraction, storage conditions, and moisture content should be reported. Where possible, ash content and representative chemical or spectroscopic fingerprints should also be determined.

This requirement is particularly important if adsorption data are to be compared between batches. A difference in Pb^2+^ uptake between two batches cannot confidently be attributed to experimental error if the pectin content, degree of esterification, mineral content, and particle-size distribution of the two raw materials are unknown.

The same principle applies to silk fibroin. “Silk fibroin” may refer to degummed fibers, dissolved and regenerated fibroin, powder, film, membrane, hydrogel, or other formats [17,18,19,20,21]. Processing alters β-sheet content, crystallinity, hydration, surface area, and accessibility of polar domains. Therefore, the physical form of fibroin should be regarded as part of the material definition rather than merely a sample-preparation detail.

For waste-derived pectin, the source should likewise remain traceable. Pectin extracted from orange peel should not automatically be treated as equivalent to commercial citrus pectin or sugar-beet pectin. If the objective is valorization of the selected wastes, the most scientifically informative comparison would be whole peel → extracted pectin → deliberately modified or crosslinked pectin derived from the same precursor. Such a matched series would allow the contribution of the pectic fraction and the effect of polymer engineering to be separated more clearly than comparisons between unrelated literature materials.

### 7.2. Raw and Modified Biosorbents Should Be Evaluated in Matched Experiments

A recurring problem in the adsorption literature is that modification is often judged by comparing a new material with capacity values reported elsewhere under different experimental conditions. This approach can substantially exaggerate the benefit of modification.

The studies discussed in Section 4 show why matched controls are preferable. Schiewer and Iqbal directly compared orange peel, depectinated peel, and extracted pectic acid and demonstrated that removal of pectin decreased Cd^2+^ binding but did not eliminate it [35]. Similarly, chemically modified orange-peel studies demonstrate considerable increases in metal uptake, but these increases can only be interpreted properly when the untreated precursor is tested under the same conditions [36,37,43].

For the proposed waste-derived biosorbents, each modification should therefore be accompanied by an untreated or minimally processed control prepared from the same batch of precursor. For banana and orange peels, a rational first comparison could include:

1. washed and dried raw peel;
2. a controlled alkaline or demethylation treatment intended to increase accessible carboxylate groups;
3. where justified, subsequent Ca^2+^ crosslinking or ionic conditioning.

This progression is preferable to immediately introducing graft polymers, synthetic chelating ligands, magnetic nanoparticles, or activated carbon because it allows the contribution of the native polymer matrix to remain visible.

The Mn^2+^ study using demethoxylated, Ca-crosslinked orange peel illustrates the value of this approach. The treatment increased the availability of COO^−^ groups and introduced exchangeable Ca^2+^ into the material, while FTIR and SEM–EDX supported an ion-exchange contribution during Mn^2+^ uptake [33]. Similarly, NaOH/CaCl_2_-modified orange peel showed substantial capacities for Cu^2+^, Pb^2+^, and Zn^2+^ and provided direct evidence for Ca^2+^ displacement [37].

For pectin, the most mechanistically informative comparison would be between the extracted polymer before and after controlled reduction in esterification and Ca^2+^ crosslinking. Earlier pectin studies already show that a lower degree of esterification increases Pb^2+^ binding and that Ca-crosslinked gels can participate directly in ion exchange [5,31].

For fibroin, aggressive chemical functionalization should not necessarily be the first step. The existing literature already demonstrates metal uptake and repeated regeneration by comparatively simple fibroin materials [7,21]. Establishing the performance of a reproducible fibroin powder, fibrous mat, or regenerated film would therefore provide a more useful baseline before adding synthetic ligands.

The central design principle is consequently that modification should be introduced to solve a demonstrated limitation, not merely to maximize q_max_. Possible limitations include insufficient binding-site density, weak mechanical stability, excessive swelling, slow kinetics, poor selectivity, or difficult separation from water. Each modification should be justified against one or more of these specific deficiencies.

### 7.3. Adsorption Mechanisms Require More than Model Fitting

A second major research gap concerns mechanistic interpretation. The widespread use of Langmuir, Freundlich, pseudo-first-order, and pseudo-second-order models has generated a large quantity of apparently mechanistic terminology that often exceeds what the experimental data demonstrate.

As discussed earlier, good agreement with pseudo-second-order kinetics does not establish chemisorption [40]. Vitek and Masini further showed that nonlinear regression is preferable to linearized isotherm transformations when estimating sorption capacity and affinity, particularly for heterogeneous materials containing more than one class of binding site [73]. Recent methodological guidance likewise emphasizes that kinetic, isotherm, thermodynamic, and fixed-bed models must be applied within their physical assumptions rather than selected solely based on a high correlation coefficient [74].

This issue is especially important for banana and orange peels because their surfaces are intrinsically heterogeneous. A mathematically acceptable Langmuir fit cannot convert a cellulose–hemicellulose–pectin–lignin matrix into a chemically homogeneous surface. Similarly, a Freundlich fit should not be treated as direct structural proof of a particular adsorption mechanism.

Future mechanistic work should therefore combine sorption measurements with independent material characterization. At minimum, comparison of the material before and after metal loading should include FTIR spectroscopy. However, small FTIR peak shifts alone should also be interpreted cautiously. Stronger evidence can be obtained when FTIR is combined with complementary methods such as:

- SEM–EDX to verify accumulation and elemental distribution;
- XPS, where available, to examine changes in surface chemical states;
- measurement of released Ca^2+^ or other counterions to test ion exchange;
- zeta potential or pH_pzc_ to establish surface-charge behavior;
- comparison of different degrees of pectin esterification;
- measurement of swelling and morphological changes after crosslinking;
- characterization before and after regeneration.

The strongest mechanistic studies reviewed here are those in which the polymer itself was deliberately altered and the consequence was subsequently measured. Examples include comparison of different pectin esterification states [31], depectination of orange peel [35], and Ca^2+^ displacement from crosslinked pectin-rich matrices [5,33,37]. For the proposed experimental programme, the mechanism should therefore be inferred from converging evidence, not from a single adsorption equation.

### 7.4. Single-Metal Solutions Are Necessary for Screening but Insufficient for Material Selection

Most biosorbent development begins with single-metal synthetic solutions. This is appropriate for identifying pH dependence, equilibrium behavior, kinetics, and approximate capacity. It is not sufficient, however, for predicting performance in environmental or industrial water.

Natural and waste waters contain major cations such as Ca^2+^, Mg^2+^, Na^+^, and K^+^, together with variable concentrations of Fe, Mn, dissolved organic matter, inorganic anions, and competing toxic metals. If several ions interact with the same carboxylate or carbonyl sites, the apparent capacity measured in a single-metal system can decrease substantially.

Pectin studies already provide evidence that relative affinity depends on polymer origin and experimental conditions. Dronnet et al. and Kartel et al. reported different metal-affinity sequences for different pectin preparations [41,42]. This variability should not be regarded as a weakness of the published studies; rather, it demonstrates that selectivity is an emergent property of the polymer structure, solution chemistry, and competing ions.

A recent review of continuous adsorption using agricultural lignocellulosic residues reaches the same conclusion: multicomponent systems are considerably more complex because competitive adsorption changes selectivity and affects breakthrough behavior [68]. Multicomponent and real-effluent column studies remain far less common than monocomponent experiments [68].

A rational experimental sequence should therefore proceed from simple to complex matrices: single ion → binary mixtures → multicomponent metal solution → major-ion-containing synthetic water → real water or wastewater.

For the present materials, Pb^2+^ and Cu^2+^ are reasonable first-line cationic targets because their interactions with pectin-rich systems are comparatively well supported. Cd^2+^ provides a useful mechanistic target for evaluating the role of pectin, while Cr(VI) should be investigated separately because its oxoanion speciation requires a fundamentally different pH and electrostatic framework. Ni^2+^, Zn^2+^, Co^2+^, Ca^2+^, Mg^2+^, and Fe species may initially be particularly valuable as competitive ions, even if they are not selected as the primary target contaminants.

Selectivity should be quantified rather than described only qualitatively. Distribution coefficients, selectivity coefficients, residual concentrations, and percentage recovery or removal under competition provide more meaningful information than a statement that one metal was “preferentially adsorbed”.

For quantitative comparison, a distribution coefficient can be reported as K_d_ = q_e_/C_e_ (with units consistent with the selected mass/volume basis), and pairwise selectivity can be expressed as αA/B = K_d_,A/K_d_,B under the same multicomponent conditions. A competition–retention ratio, Rcomp = q_e_,mix/q_e_,single × 100%, is also useful for showing how much single-ion uptake is preserved in the presence of co-ions. These quantities distinguish intrinsic affinity from an apparently high percentage removal that may simply reflect a high sorbent dose or a low initial concentration. When selectivity is claimed, the initial concentrations, ionic strength, pH, dose, equilibrium residual concentrations, and uncertainty of K_d_ or α should be reported explicitly.

### 7.5. Environmental Concentrations Should Be Included Alongside High-Concentration Isotherms

Adsorption-capacity studies frequently use metal concentrations chosen to obtain well-defined isotherms and measurable saturation. Such experiments are scientifically useful, but they may operate far above the concentrations encountered in treated wastewater or environmental monitoring. This creates an important gap between capacity research and practical removal.

A sorbent may show a very high q_max_ at tens or hundreds of milligrams per liter yet leave an unacceptable residual metal concentration when the starting concentration is much lower. Conversely, a lower-capacity material may have a high distribution coefficient at low concentration and therefore perform better in polishing applications.

Reviews of agro-waste biosorption repeatedly identify insufficient testing with real wastewater and realistic contaminant concentrations as a major obstacle to translation [68,70,71,72]. Parida et al. emphasize the need for studies that integrate realistic wastewater matrices, regeneration, and practical material handling before agro-waste biosorbents can progress reliably toward large-scale use [70].

Future studies should therefore include two complementary concentration regimes:

- a sufficiently broad range for equilibrium modeling and estimation of sorption capacity;
- a low-concentration range relevant to the intended treatment or analytical application.

Reporting only percentage removal is also insufficient. The equilibrium residual concentration should be reported because a nominal 95–99% removal can still leave an environmentally significant concentration when the initial concentration is very high.

### 7.6. Batch Adsorption Should Be Followed by Continuous-Flow Evaluation

Batch experiments are indispensable for early material development, but a practical water-treatment biosorbent will usually need to function under flowing conditions. The gap between batch and continuous operation is substantial. In a fixed-bed column, mass transfer, residence time, bed depth, flow rate, pressure drop, particle stability, channeling, and breakthrough behavior become important. A material that disperses easily in batch experiments may pack poorly in a column. Fine particles that provide rapid adsorption may generate an unacceptable hydraulic resistance. Strongly swelling pectin gels may alter bed permeability during operation.

The recent Polymers review by Coelho et al. is particularly relevant because it evaluates raw and modified agricultural lignocellulosic residues specifically in continuous adsorption systems [68]. The review identifies scale-up of laboratory modification routes, multicomponent competition, and translation to continuous operation as central challenges for technology development [68].

For the four matrices considered here, continuous-flow testing should therefore be regarded as a later but essential development stage. Raw fruit-peel powders may require granulation, immobilization, or controlled particle-size selection before column use. Extracted pectin may require formation of mechanically stable beads, films, foams, or supported structures. Fibroin has a potential advantage because it can be processed into fibers, mats, membranes, particles, and other self-supporting architectures [17,18].

Column development should begin only after the batch experiments have identified the most promising material–metal combinations. The first continuous experiments should determine:

- breakthrough curves;
- dynamic adsorption capacity;
- bed utilization;
- influence of flow rate;
- influence of bed height;
- regeneration performance within the packed bed;
- mechanical stability after repeated loading and elution.

Where possible, both single-metal and multicomponent breakthrough curves should be obtained.

### 7.7. Regeneration Should Preserve Material Function, Not Only Remove the Metal

Section 6 has already shown that regeneration must be distinguished from desorption. This distinction becomes even more important when defining research priorities. Fibroin reportedly retained bulk sorption performance after repeated EDTA-assisted cycles [7], while Ca-crosslinked pectin materials have been regenerated through acid desorption followed by restoration of the Ca^2+^ form [26]. These examples are promising, but the criteria for a practical biosorbent should be stricter.

After each regeneration cycle, at least the following should be considered:

- adsorption capacity or removal performance;
- equilibrium time;
- material mass loss;
- mechanical integrity;
- FTIR or other evidence of chemical stability;
- morphology;
- leaching of polymer components or modification reagents;
- composition and volume of the desorption solution.

A material that retains 90% of its original adsorption capacity but loses a substantial fraction of its mass after each cycle is not truly stable. Similarly, successful acid desorption is not sufficient if repeated acid exposure gradually changes pectin esterification, hydrolyzes the matrix, or alters fibroin structure.

Recent literature emphasizes that long-term stability, recovery of the retained metal, secondary waste, and final management of the exhausted biosorbent remain incompletely resolved [61,69]. Mileta and Nuić argue for a full-cycle approach extending from adsorption to regeneration and safe end-of-life handling of metal-loaded biosorbents [69]. The desorbed metal should also be considered a potentially recoverable resource rather than simply an analytical waste. Whether recovery is economically meaningful will depend on metal identity and concentration, but measuring the mass balance through adsorption and desorption is essential.

### 7.8. End-of-Life Management Must Be Included from the Beginning

Waste-derived biosorbents are frequently described as biodegradable, renewable, or environmentally benign. These terms are appropriate for the precursor but may not remain appropriate for the exhausted material. After sorption, the biosorbent contains concentrated Pb, Cd, Cr, Cu, Ni, or other metals. Uncontrolled biodegradation, composting, or disposal can therefore release the retained contaminants and transfer the environmental problem rather than resolve it.

This issue has recently received greater attention. Reviews of metal-loaded adsorbents emphasize regeneration, metal recovery, controlled reuse of exhausted materials, and stabilization before disposal as alternatives to uncontrolled release [61,69,75]. Fouda-Mbanga et al. specifically identify the fate of metal-loaded adsorbents as a major issue because inappropriate disposal can create secondary pollution [75].

For the present project, end-of-life considerations need not become a major experimental programme at the initial screening stage, but they should influence material selection. A biosorbent that requires hazardous reagents for manufacture, cannot be regenerated, and generates an unstable metal-rich residue may be less attractive than a somewhat lower-capacity material that is easily regenerated.

Accordingly, the comparison of candidate materials should ultimately include not only q_max_ but also capacity + selectivity + kinetics + regeneration + stability + preparation burden + metal recovery + final waste management.

### 7.9. Biosorption and Optical Sensing Should Be Developed Sequentially

The optical concept discussed in Section 5 remains scientifically interesting, but the literature reviewed here suggests that it should be treated as a second-stage functionalization objective rather than as the primary criterion for selecting a biosorbent. The correct sequence is to first establish a reproducible sorbent.

Only after the material has a defined particle size or geometry, reproducible chemical composition, sufficiently stable metal uptake, and acceptable regeneration behavior should its optical response be evaluated. This sequence avoids an important methodological problem. If different batches of raw peel differ in composition, particle size, moisture, and packing behavior, changes in diffuse reflectance may arise from the material itself rather than metal loading. A calibration obtained under such conditions could appear statistically acceptable while lacking transferability between batches.

The initial optical experiment should therefore not begin with calculation of a detection limit. Instead, full reflectance spectra should be compared for:

1. unloaded biosorbent;
2. biosorbent exposed to increasing amounts of target metal;
3. regenerated biosorbent;
4. biosorbent exposed to competing ions;
5. blank materials subjected to the same pH, washing, and drying treatment.

Only if a reproducible metal-dependent spectral change is observed should Kubelka–Munk transformation, wavelength selection, univariate calibration, or multivariate modeling be attempted. Existing solid-phase spectroscopic studies demonstrate that polymer-supported metal analysis is technically feasible [47,48,49,50,51,58]. A recent silk-fibroin-based Pb(II) method using methyl thymol blue immobilized on fibroin further demonstrates the feasibility of combining a natural-polymer sorption phase with direct spectrophotometric readout [76]. However, the more difficult question remains whether a minimally modified waste-derived polymer can generate an intrinsic or sufficiently stable metal-dependent signal. This is best regarded as a value-added application of an already validated biosorbent, rather than evidence required to establish the biosorbent itself.

### 7.10. Statistical Treatment and Adsorption Modeling Should Be Strengthened

Future experiments should also improve the statistical quality of adsorption data. Replicate measurements and uncertainty should accompany equilibrium and kinetic data. Model selection should not rely only on R^2^, especially when comparing models with different numbers of parameters. Nonlinear regression should generally be preferred to linearization of nonlinear isotherm equations because transformation can change error structure and bias fitted capacity and affinity parameters [73].

Vitek and Masini showed explicitly that nonlinear analysis provides more reliable adsorption parameters than linearized Langmuir treatment and is especially important for heterogeneous sorbents with multiple site classes [73]. More general modeling guidance recommends matching each model to its physical assumptions and using appropriate error or information criteria rather than treating the model with the highest correlation coefficient as mechanistically correct [74].

Accordingly, future studies should report:

- experimental points rather than fitted curves alone;
- replicate variability or uncertainty;
- nonlinear model parameters with appropriate errors;
- residual analysis;
- explicit model-selection criteria;
- distinction between experimental q_e_ and model-derived q_max_;
- avoidance of mechanistic claims based only on kinetic-model identity.

For heterogeneous waste-derived materials, this statistical discipline is particularly important because real variability in the adsorbent may otherwise be hidden behind apparently excellent mathematical fits. This emphasis is consistent with the broader cellulose-based sorbent literature, which highlights surface functionalization, chemical modification, morphology, and regeneration as interdependent design variables in water-remediation materials [77].

### 7.11. Proposed Design Rules for the Four Waste-Derived Platforms

**Silk fibroin**

Silk-processing waste should first be converted into a reproducible fibroin form with controlled processing history. A minimally modified powder, mat, or film should be characterized before considering chelating functionalization. The main advantages to preserve are structural processability and demonstrated reusability [7,17,18,21]. Pb^2+^ and Cu^2+^ are useful initial cationic targets, whereas chromate should be treated separately because of its anionic speciation.

**Banana peel**

The priority should be standardization of precursor variety, drying, and particle size. The raw peel should be compared with one controlled modification rather than a large range of chemically unrelated treatments. Because extracted banana pectin can be highly esterified [4], controlled de-esterification may be a rational route if the aim is to increase accessible carboxylate sites. Pb^2+^, Cd^2+^, and Cu^2+^ provide suitable initial targets, with Ca^2+^/Mg^2+^ competition included at a later stage.

**Orange peel**

Orange peel provides the clearest literature basis for examining the role of pectin because raw peel, depectinated peel, extracted pectic material, and Ca-conditioned materials have all been studied [30,33,35,37]. A particularly informative experimental series would therefore be raw orange peel → mildly de-esterified peel → Ca^2+^-conditioned peel. This would directly test whether increasing carboxylate accessibility and creating exchangeable Ca^2+^ sites justify the additional preparation step.

**Waste-derived pectin**

Pectin isolated from the same fruit residues should be treated as a separate polymer platform. Degree of esterification should be measured rather than assumed. Comparison of non-crosslinked and Ca-crosslinked pectin can establish the contribution of network formation and ion exchange. Mechanical stability and swelling should be evaluated together with adsorption capacity because these properties will determine whether the material can be recovered, regenerated, and reused without unacceptable loss of mass or function.

The main research gaps and recommended experiments are summarized in Table 6.

### 7.12. Recommended Experimental Roadmap

Based on the critical evidence reviewed in this article, a staged experimental strategy is more defensible than simultaneous optimization of every material and metal.

**Stage I—precursor selection and standardization.** Collect and document silk-processing residue, banana peel, orange peel, and the selected pectin source. Establish reproducible washing, drying, milling, storage, and, where applicable, fibroin isolation or pectin extraction procedures.

**Stage II—baseline material characterization.** Characterize the untreated materials by the methods relevant to their structure. FTIR should be considered essential. SEM/EDS, XRD, thermal analysis, surface charge, swelling, porosity, or BET measurements can be selected according to material form and expected mechanism rather than applied mechanically to every sample.

**Stage III—minimal rational modification.** Select a maximum of one or two modifications initially. For fruit peels and pectin-rich materials, controlled de-esterification or alkaline treatment and Ca^2+^ conditioning are mechanistically justified by the existing literature [5,31,33,37]. For fibroin, first establish the performance of a reproducible, minimally modified material before introducing additional ligands.

**Stage IV—metal screening.** Screen representative metal ions under matched conditions. Pb^2+^ and Cu^2+^ should be included because of their well-supported affinity toward carboxylate-rich matrices. Cd^2+^ can provide additional mechanistic information. Cr(VI) should be investigated separately from cationic metals and not included blindly in a common optimization design. The need to treat oxyanionic chromium separately is also consistent with broader adsorption studies on modified bentonite systems, where bichromate uptake was governed by sorbent surface chemistry and solution conditions [78].

**Stage V—optimization and mechanism.** For the most promising material–metal pairs, determine pH dependence, dose, concentration dependence, kinetics, equilibrium, temperature if scientifically justified, and competing-ion effects. Use nonlinear modeling and direct mechanistic characterization rather than relying on kinetic-model identity alone [40,73,74].

**Stage VI—mixed-ion selectivity.** Challenge the most promising materials with controlled binary and multicomponent ion mixtures under the same pH, dose, contact time, and concentration basis used for single-metal screening. Quantify competition using K_d_, pairwise selectivity factors, and the competition–retention ratio (Rcomp), rather than percentage removal alone.

**Stage VII—regeneration and multicycle performance.** Select an eluent on the basis of both desorption efficiency and preservation of polymer structure. Record adsorption and desorption mass balances, capacity retention, dry-mass recovery, chemical/structural stability, metal recovery, and changes in material integrity over repeated cycles.

**Stage VIII—real-water validation.** Transfer the best-performing material–metal combinations from controlled mixtures to representative natural water, industrial water, or wastewater. Report the matrix composition, residual target-metal concentration, performance retention relative to the matched synthetic matrix, replicate variability, and any pretreatment used.

**Stage IX—continuous-flow evaluation.** Convert the most promising material into a geometry suitable for a column or flow-through system and determine breakthrough characteristics. This stage is essential for treatment-oriented applications because the current literature still contains limited continuous and scale-up evidence for waste-derived biosorbents [68].

**Stage X—optional solid-phase optical screening.** Only after material reproducibility, selectivity, regeneration, and matrix performance have been established should metal-dependent diffuse-reflectance changes be examined. If a robust spectral response is observed, the material may be developed further as a dual-function sorbent–sensing platform. This final stage is a value-added extension rather than a prerequisite for successful waste valorization.

Recent 2026 primary studies reinforce these design priorities while also illustrating why material classes must remain separated. Lei et al. engineered a cellulose-nanofiber/MOF/MXene composite membrane for Cr(VI), reporting a maximum adsorption capacity of 17.79 mg g^−1^, nearly complete visible-light Cr(VI) reduction, and >70% removal efficiency after 20 regeneration cycles; this highly engineered membrane is therefore informative for durability and interfacial design but is not a direct comparator for raw fruit peel or isolated pectin [79]. Peng et al. showed that low-methoxyl pectin extracted from sunflower heads combined a low degree of esterification (26.34%) with accessible carboxyl groups and achieved a Pb^2+^ adsorption capacity of 295.86 mg g^−1^, supported by FTIR, XPS, SEM–EDS, BET, and competitive-adsorption evidence [80]. Rahman et al. evaluated untreated banana and orange peels for Cu(II), Pb(II), and total dissolved Cr under controlled batch conditions and demonstrated strong pH dependence, but also identified regeneration, isotherm/kinetic validation, and spent-sorbent management as necessary next steps [81]. These recent studies strengthen the structure–property–performance and translation framework used here without justifying numerical pooling across different material forms.

To convert the roadmap into a decision tool, each stage is linked below to an explicit operational go/no-go gate. These thresholds are proposed screening criteria for research progression, not universal regulatory acceptance limits; they should be replaced by stricter application-specific targets when drinking-water, discharge, or analytical regulations require them. The complete evidence-based roadmap for the development of waste-derived natural-polymer biosorbents is summarized in Figure 6.

The proposed quantitative stage-gates for progression through the experimental roadmap are summarized in Table 7.

## 8. Conclusions

Silk fibroin, banana peel, orange peel, and pectin represent chemically distinct but complementary natural-polymer platforms for the development of waste-derived biosorbents. Their comparison shows that metal-ion sorption is governed not simply by precursor origin or the nominal presence of functional groups, but by the accessibility and chemical state of those groups, polymer organization, morphology, processing history, and solution conditions.

Banana and orange peels are attractive because they are inexpensive and readily available agro-industrial residues containing pectin, cellulose, hemicellulose, lignin, and other minor components. Their heterogeneous composition, however, is also their principal limitation. Reliable comparison of these materials therefore requires careful standardization of precursor source, washing, drying, particle size, storage, and chemical pretreatment. Without such control, differences in sorption performance may reflect variability of the biomass rather than a true material effect.

Pectin offers a greater degree of chemical control. Its galacturonic-acid-rich structure provides carboxyl groups whose availability can be adjusted through esterification and ionic crosslinking. Lower esterification generally increases the population of accessible carboxylate sites, while Ca^2+^ crosslinking can introduce both structural stabilization and an ion-exchange pathway. The resulting sorption behavior is therefore determined by a balance between functional-group density, crosslinking, swelling, porosity, and mechanical stability rather than by adsorption capacity alone.

Silk fibroin provides a structurally different protein-based platform. Its metal-binding behavior is associated with peptide carbonyl groups, hydroxyl-containing residues, ionizable side groups, and the accessibility of hydrated regions of the protein matrix. Its ability to be processed into fibers, films, powders, and other forms, together with its demonstrated reusability, makes fibroin particularly interesting for the development of mechanically stable and regenerable biosorbent materials.

The reviewed evidence also demonstrates that a universal ranking of these four materials is not scientifically justified. Reported capacities are strongly dependent on pH, initial concentration, sorbent dose, particle size, modification, esterification, crosslinking, and the selected equilibrium model. Consequently, model-derived q_max_, experimentally measured adsorption capacity, and removal percentage should not be treated as interchangeable indicators of material performance. Likewise, kinetic or isotherm fitting alone is insufficient to establish a molecular adsorption mechanism.

From a materials-design perspective, Pb^2+^ and Cu^2+^ appear to be suitable initial targets for carboxylate-rich biosorbents, while Cd^2+^ is particularly useful for evaluating the contribution of pectin. Chromium should be considered separately because Cr(VI) oxoanions and Cr(III) cations differ fundamentally in aqueous speciation and interaction with polymer surfaces. Other metal ions, including Ni^2+^, Co^2+^, Zn^2+^, Ca^2+^, Mg^2+^, and Fe species, are especially important for evaluating competitive sorption and selectivity under realistic conditions.

The major challenge for future work lies in moving beyond first-cycle batch adsorption. Reproducible precursor preparation, matched comparison of untreated and modified materials, multicomponent testing, real-water evaluation, regeneration, continuous-flow operation, and safe management of metal-loaded biosorbents should become integral parts of material development. The objective should not be to obtain the highest possible adsorption capacity through increasingly complex modification, but to identify the minimum rational modification required to achieve sufficient capacity, selectivity, stability, and reusability.

Regeneration is particularly important because a practical biosorbent must preserve not only its adsorption capacity but also its chemical functionality, morphology, and mechanical integrity during repeated use. Desorption should therefore be evaluated together with restoration of sorption performance, metal recovery, mass loss, and the final management of the exhausted polymer. This full-cycle perspective is essential if the environmental advantages of waste-derived materials are to be preserved.

Diffuse-reflectance sensing may provide an additional functional direction for these biosorbents, but it should be considered only after the sorption material itself has been standardized. Stable optical measurement requires reproducible particle size or material geometry, hydration state, surface morphology, thickness, and scattering behavior. Therefore, sensing should be viewed as a value-added extension of a validated biosorbent rather than as the primary criterion for material selection.

Overall, the most promising strategy is to transform waste-derived natural polymers from variable biological residues into deliberately controlled functional materials. A common experimental framework—precursor standardization → controlled modification → structure–property characterization → metal sorption and selectivity → regeneration → real-water and continuous-flow validation → optional sensing functionality—would allow the four platforms to be compared on a scientifically consistent basis. Such an approach provides a rational pathway for converting food-processing and silk-related wastes into reproducible, selective, regenerable, and practically relevant biosorbents for water treatment and environmental applications.

## Figures and Tables

**Figure 1:**
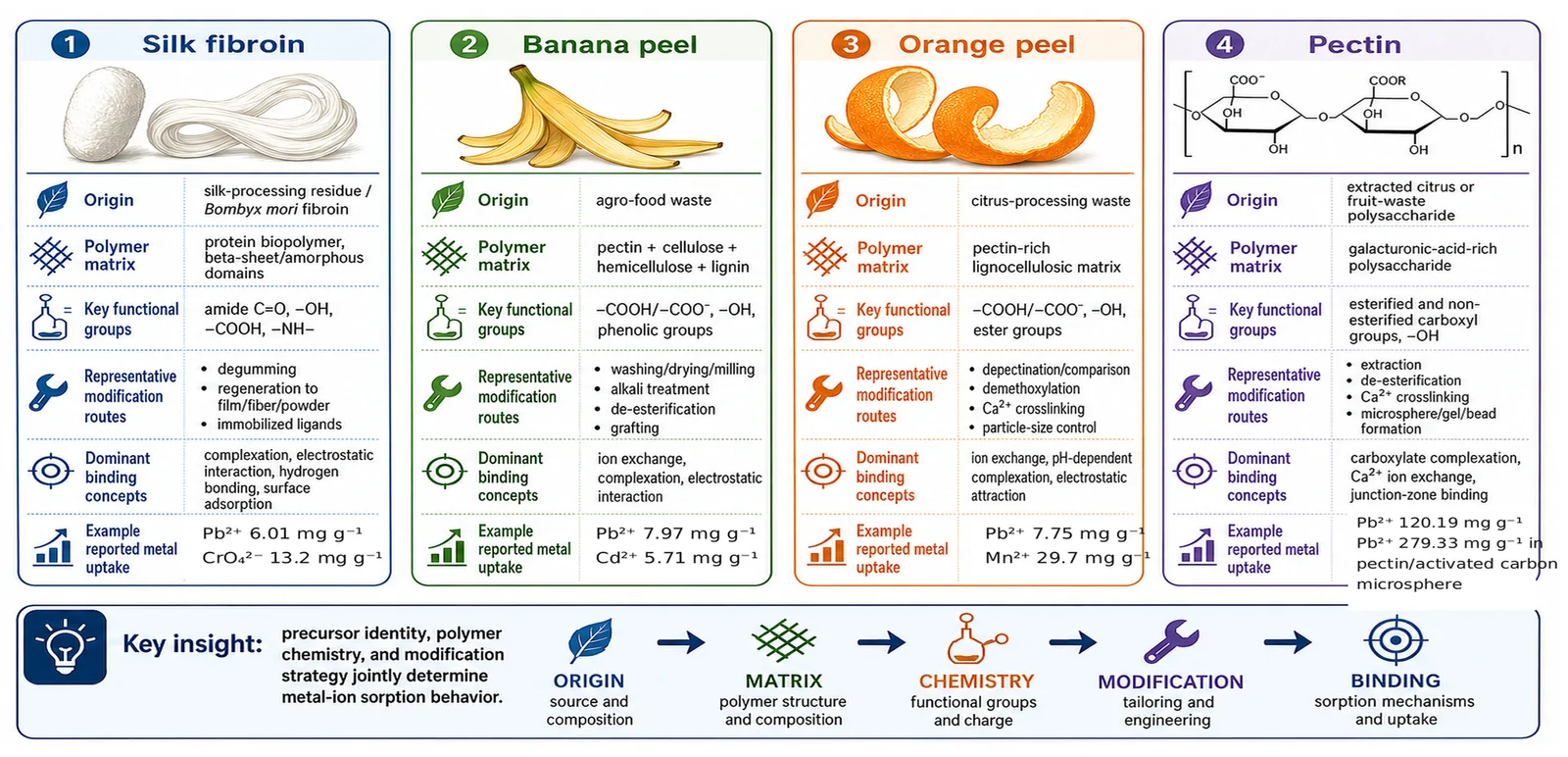
The four biosorbent precursor platforms: waste/biological origin, polymer-matrix type, principal functional-group chemistry, representative modification routes, and dominant metal-binding concepts. Representative literature data shown in the figure are based on Refs. [2,7,16,17,18].

**Figure 2:**
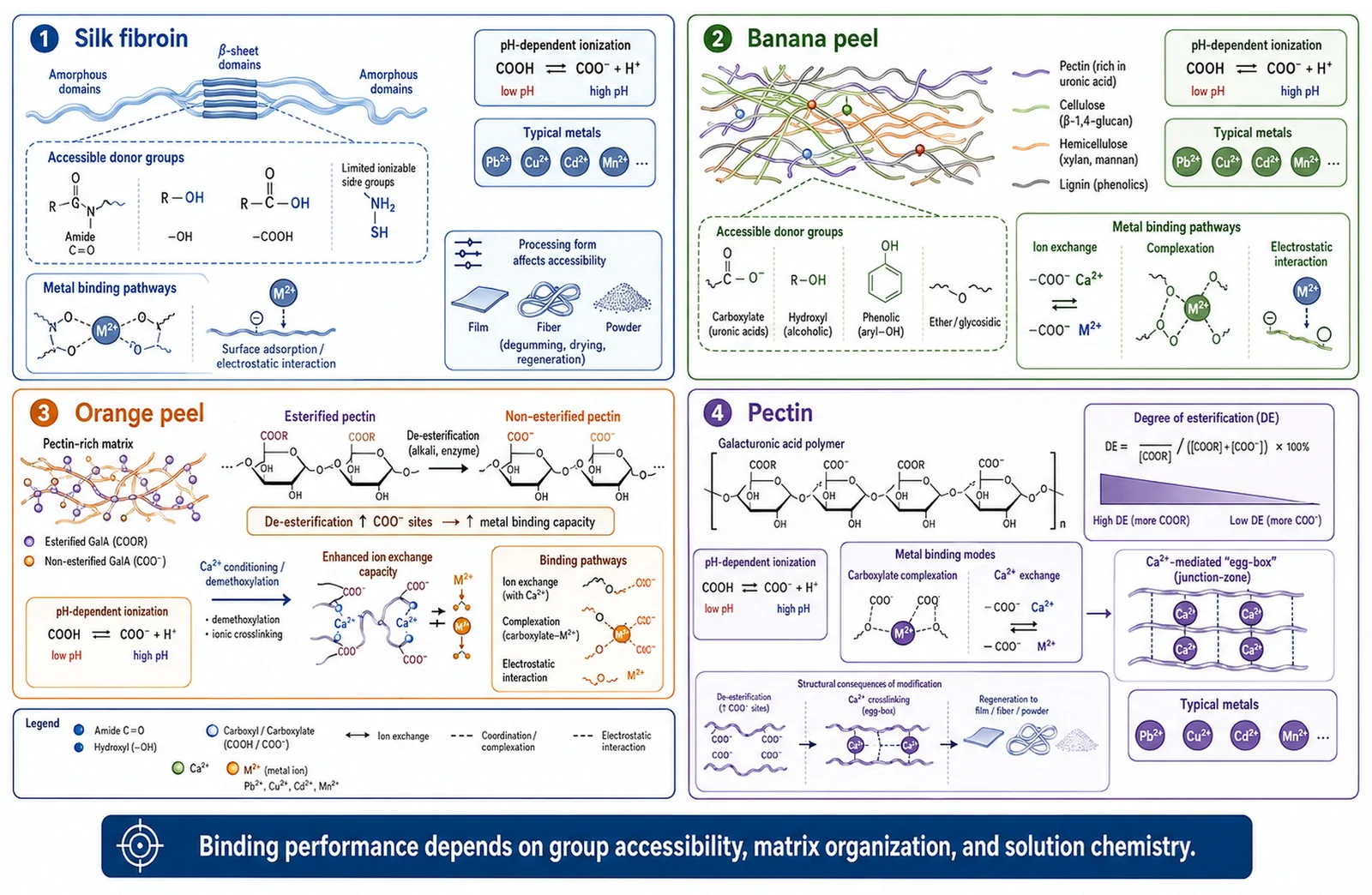
Structure–metal binding relationships in silk fibroin, banana peel, orange peel, and pectin, emphasizing accessible donor groups, carboxylate ionization, ion exchange, Ca^2+^-mediated junction zones, and the structural consequences of modification. Colors distinguish the four biosorbent platforms; arrows indicate the principal ionization, ion-exchange, coordination/complexation, electrostatic-interaction, and structural-modification pathways.

**Figure 3:**
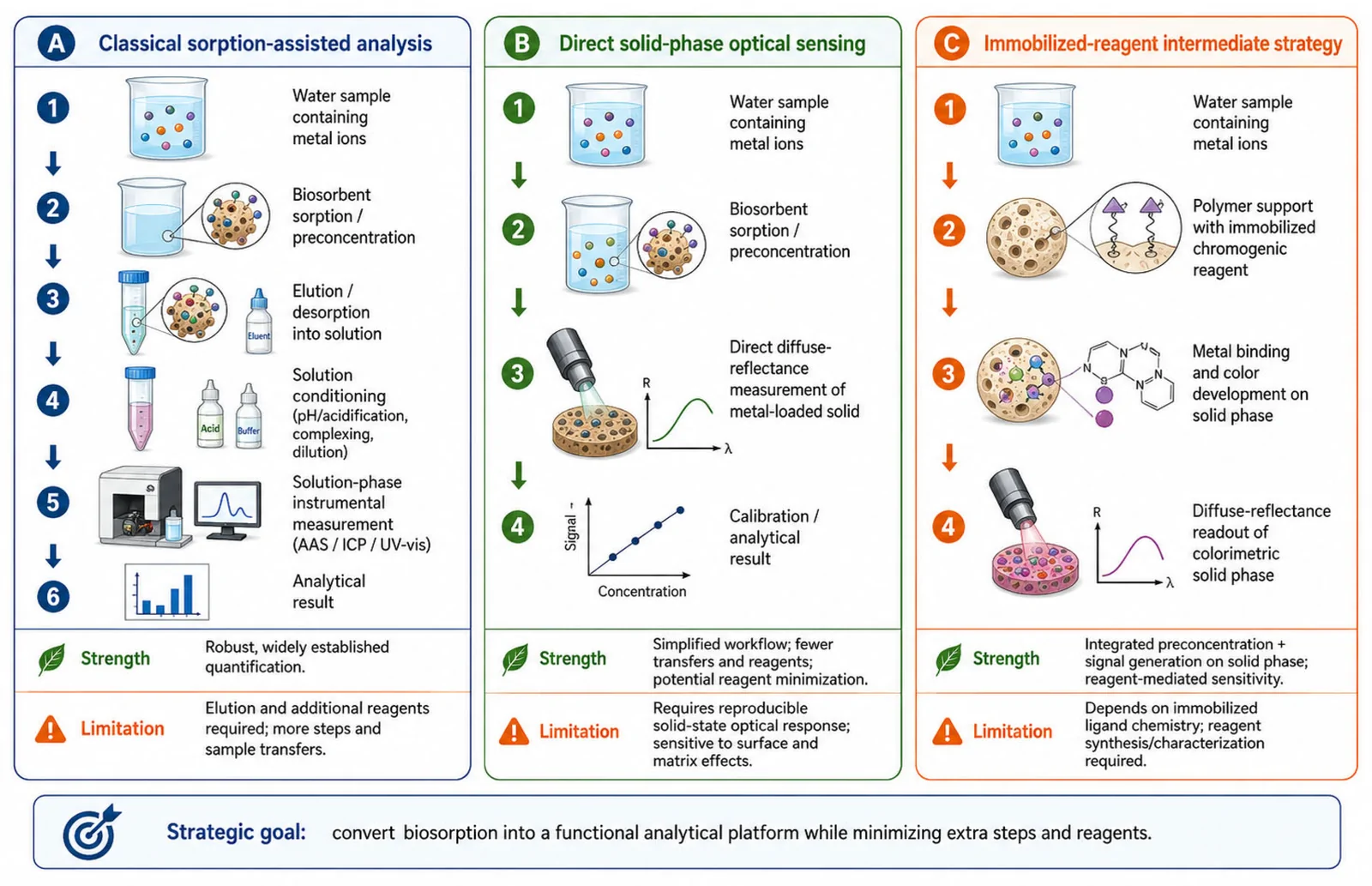
Classical and direct solid-phase analytical pathways. The conventional route includes sorption followed by elution and solution-phase measurement; the direct route seeks optical readout from the metal-loaded solid. Immobilized-reagent systems represent an intermediate strategy.

**Figure 4:**
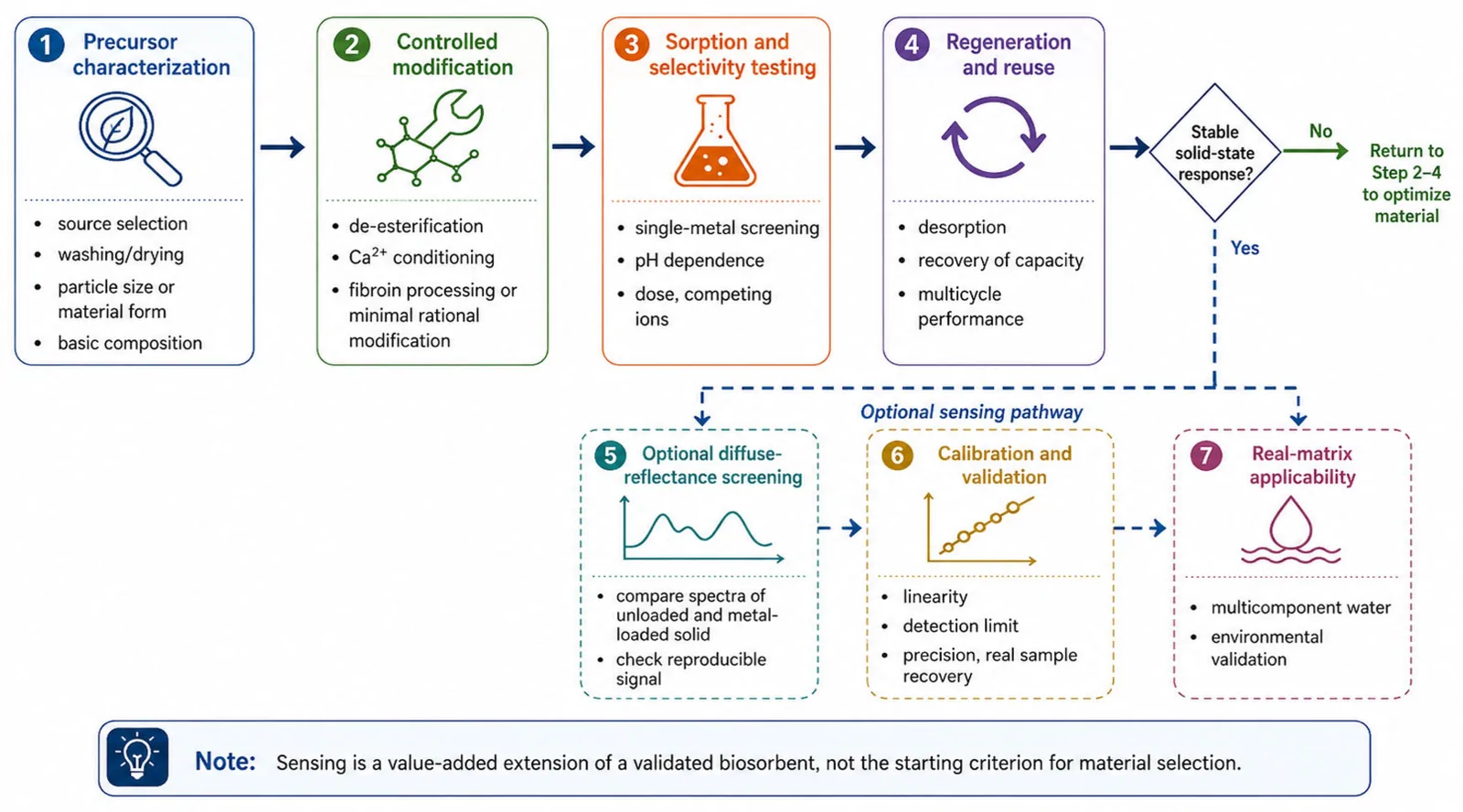
Proposed staged pathway from waste-derived biosorbent development to optional sensing: precursor characterization → controlled modification → sorption/selectivity testing → regeneration and reuse → optional diffuse-reflectance screening → calibration and real-matrix validation.

**Figure 5:**
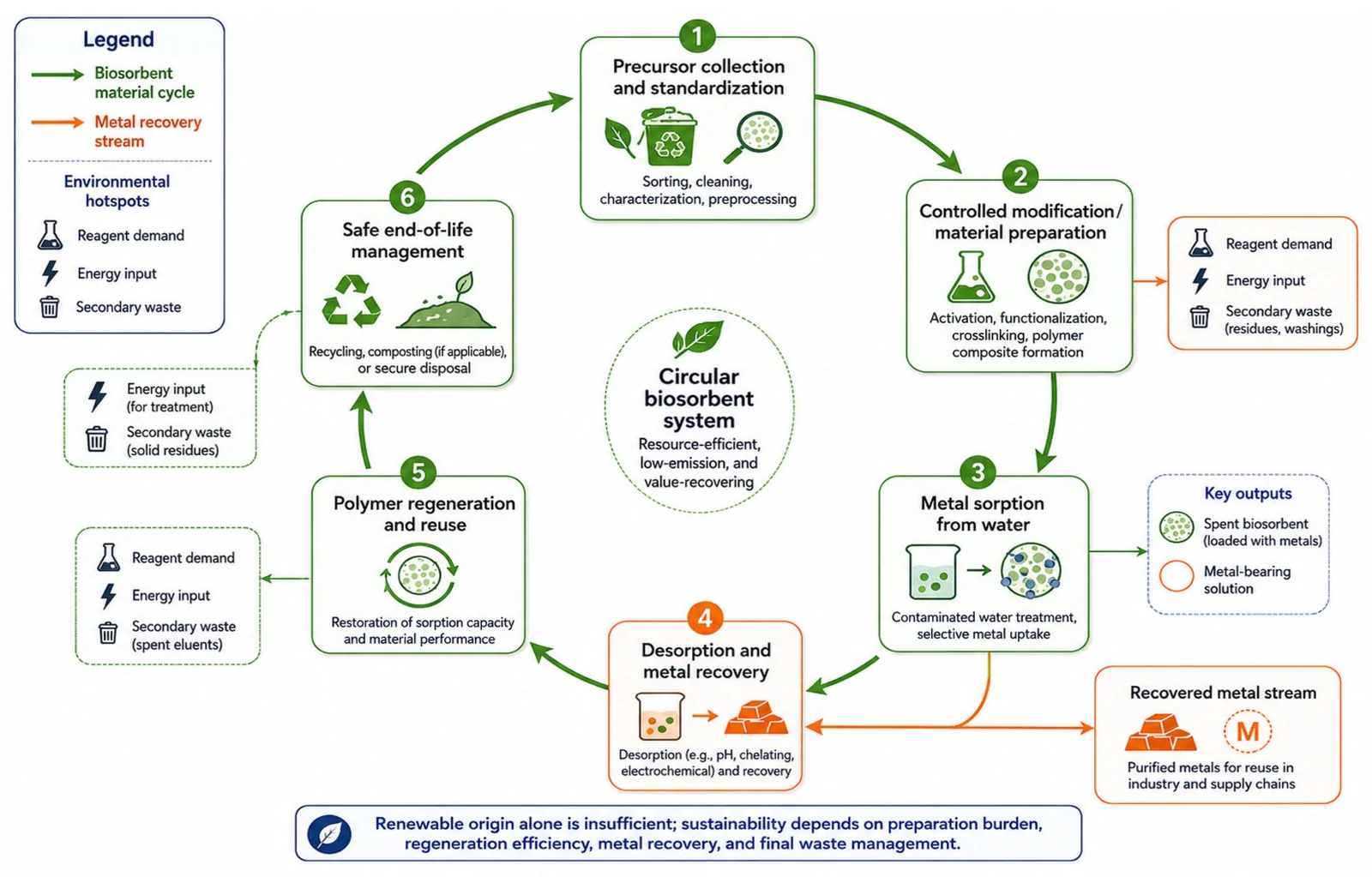
Proposed circular development pathway for a waste-derived biosorbent: precursor collection and standardization → controlled modification → metal sorption → desorption/metal recovery → polymer regeneration and reuse → safe end-of-life management. The pathway explicitly distinguishes material reuse from metal recovery and identifies preparation and regeneration as stages that contribute to reagent and energy demand.

**Figure 6:**
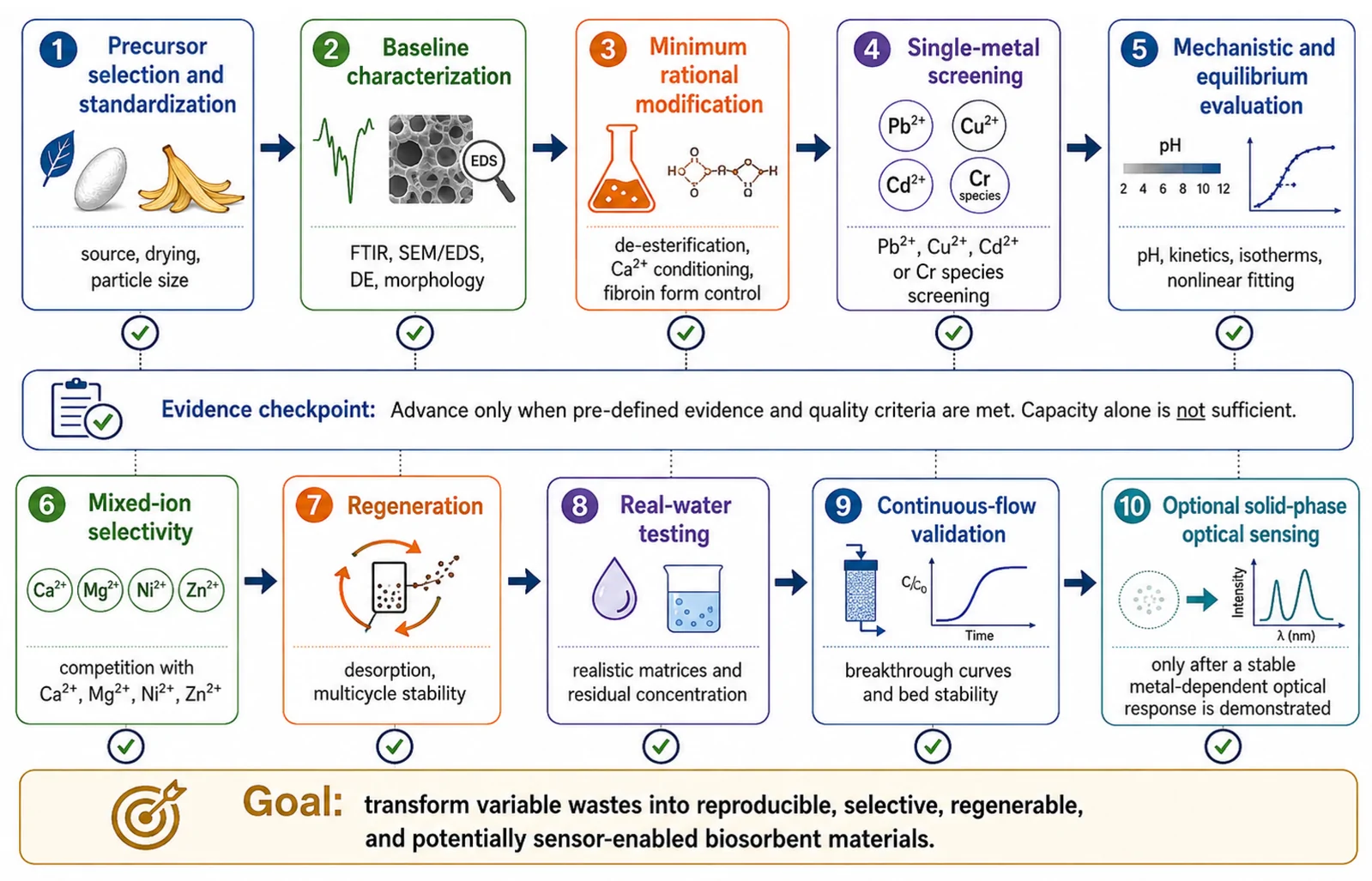
Evidence-based roadmap for development of waste-derived natural-polymer biosorbents. Suggested sequence: precursor selection and standardization → baseline characterization → minimum rational modification → single-metal screening → mechanistic and equilibrium evaluation → mixed-ion selectivity → regeneration → real-water validation → continuous-flow validation → optional solid-phase optical sensing. Quantitative go/no-go criteria for Stages I–X are provided in Table 7. The proposed thresholds are research-stage decision gates rather than universal regulatory acceptance criteria. Arrows indicate the sequential progression through the proposed development stages; check marks denote evidence checkpoints that should be satisfied before progression to the next stage. Colors distinguish the development modules and do not represent quantitative values.

**Table 1:** Standardized, source-traceable adsorption data for the waste-derived and natural polymer-based biosorbents considered in this review.

Polymer Matrix/Material and Standardized Class Composition Descriptor	Metal Ion	Material Form/Modification and Key Test Conditions	Reported Adsorption Metric (Metric Type Stated)	Selectivity, Regeneration, and Mechanistic Evidence	Ref.
Isolated protein biopolymer Silk fibroin Composition: protein matrix (semicrystalline fibroin polypeptide)	Pb^2+^	Fibroin extracted from *Bombyx mori* cocoon. C_0_/pH/dose: not tabulated in the review; see cited source.	Source-reported sorption capacity: 6.01 mg g^−1^ (not pooled with model-derived q_max_ from other studies).	Sorption isotherms and thermodynamic analysis; sorption classified by the authors as physical, spontaneous, and endothermic	[7]
Isolated protein biopolymer Silk fibroin Composition: protein matrix (semicrystalline fibroin polypeptide)	CrO_4_^2−^	Fibroin extracted from *Bombyx mori* cocoon. C_0_/pH/dose: not tabulated in the review; see cited source.	Source-reported sorption capacity: 13.2 mg g^−1^.	Anionic chromate must be considered separately from divalent cations; thermodynamic and kinetic characterization reported	[7]
Isolated protein biopolymer Silk fibroin Composition: protein matrix (semicrystalline fibroin polypeptide)	Cu^2+^	Fibroin extracted from *Bombyx mori* cocoon. C_0_/pH/dose: not tabulated in the review; see cited source.	Source-reported sorption capacity: 2.29 mg g^−1^.	Sorption characterized together with Pb^2+^, CrO_4_^2−^ and Co^2+^; regenerated with EDTA	[7]
Isolated protein biopolymer—reuse test Silk fibroin Composition: protein matrix (semicrystalline fibroin polypeptide)	Pb^2+^, CrO_4_^2−^, Cu^2+^, Co^2+^	Repeated sorption–desorption; EDTA-assisted regeneration; sorption conditions as in the cited study.	Multicycle retention: no measurable loss of sorption capacity over 10 cycles.	EDTA stripping demonstrated substantial reusability of the fibroin phase	[7]
Raw polymer-rich biomass Banana peel Composition: plant cell-wall matrix containing pectin/cellulose/hemicellulose/lignin	Pb^2+^	Banana-peel sorbent; C_0_ = 5–25 mg L^−1^; 30 °C; reported Pb^2+^ value at pH 5.5.	Condition-specific equilibrium uptake: up to 7.97 mg g^−1^ for Pb^2+^ at pH 5.5.	Uptake increased with pH; zeta-potential changes supported involvement of surface charge	[2]
Raw polymer-rich biomass Banana peel Composition: plant cell-wall matrix containing pectin/cellulose/hemicellulose/lignin	Pb^2+^, Ni^2+^, Zn^2+^, Cu^2+^, Co^2+^	Same matched comparative study as row above; C_0_ = 5–25 mg L^−1^; 30 °C; pH investigated.	Condition-specific uptake: 7.97, 6.88, 5.80, 4.75, and 2.55 mg g^−1^ for Pb^2+^, Ni^2+^, Zn^2+^, Cu^2+^, and Co^2+^.	Affinity order under comparable conditions: Pb^2+^ > Ni^2+^ > Zn^2+^ > Cu^2+^ > Co^2+^	[2]
Raw polymer-rich biomass Orange peel Composition: plant cell-wall matrix containing pectin/cellulose/hemicellulose/lignin	Pb^2+^, Ni^2+^, Zn^2+^, Cu^2+^, Co^2+^	Same matched comparative study as banana peel; C_0_ = 5–25 mg L^−1^; 30 °C; pH investigated.	Condition-specific uptake: 7.75, 6.01, 5.25, 3.65, and 1.82 mg g^−1^ for Pb^2+^, Ni^2+^, Zn^2+^, Cu^2+^, and Co^2+^.	Same affinity order as banana peel under the conditions investigated	[2]
Raw polymer-rich biomass Banana peel Composition: plant cell-wall matrix containing pectin/cellulose/hemicellulose/lignin	Cd^2+^	Dried banana-peel powder; batch adsorption; pH, dose, contact time, and agitation optimized; exact settings are source-specific.	Langmuir model-derived q_max_ = 5.71 mg g^−1^ (Cd^2+^).	FAAS used for metal determination; pH, dose, contact time and agitation investigated	[29]
Raw polymer-rich biomass Banana peel Composition: plant cell-wall matrix containing pectin/cellulose/hemicellulose/lignin	Pb^2+^	Dried banana-peel powder; same batch study as Cd^2+^; exact optimized settings are source-specific.	Langmuir model-derived q_max_ = 2.18 mg g^−1^ (Pb^2+^).	Same experimental study as Cd^2+^; banana peel exhibited higher capacity for Cd^2+^ than Pb^2+^ under these conditions	[29]
Heterogeneous citrus biomass/protonated pectin-rich material Orange/lemon peel and protonated pectin-rich peel Composition: pectin-rich citrus cell-wall matrix with cellulose/hemicellulose/lignin	Cd^2+^	Citrus-derived biosorbents; pH-dependent study; proton competition explicitly modeled.	Langmuir model-derived capacity range = 0.7–1.2 meq g^−1^ (approximately 39–67 mg g^−1^ Cd^2+^).	Potentiometric titration and pH-dependent modeling supported proton competition at acidic binding sites	[30]
Isolated biopolymer preparations Low- and high-esterified pectin; calcium pectate Composition: galacturonic-acid-rich polysaccharide (pectin)	Pb^2+^	High- and low-esterified pectin and calcium pectate; batch sorption; strongest Pb^2+^ binding at approximately pH 7–8.	Relative binding result: low-esterified pectin showed the highest Pb^2+^ binding; maximum near pH 7–8; no single cross-study q_max_ used.	Direct comparison demonstrated the importance of degree of esterification	[31]
Ionically crosslinked isolated biopolymer Demethylated Ca-crosslinked sugar-beet pectin gels Composition: galacturonic-acid-rich polysaccharide (pectin)	Cd^2+^, Pb^2+^, Cu^2+^	Demethylated sugar-beet pectin hydrogel/xerogel beads crosslinked with CaCl_2_; source-specific batch conditions.	Kinetic/affinity result: kinetic uptake Cu > Pb > Cd; Langmuir affinity constants Pb ≫ Cu > Cd; no single pooled capacity used.	FTIR, SEM and Ca displacement supported ion exchange with Ca^2+^ and interaction with carboxyl groups	[5]
Ionically crosslinked isolated biopolymer Pectin microsphere Composition: galacturonic-acid-rich polysaccharide (pectin)	Pb^2+^	Ca-containing pectin microsphere; source-specific batch conditions.	Langmuir model-derived q_max_ = 120.19 mg g^−1^.	Provides a polymer-only reference for comparison with activated–carbon-containing microspheres	[32]
Polymer–carbon composite network Pectin/activated–carbon microsphere Composition: galacturonic-acid-rich pectin + activated carbon	Pb^2+^	Pectin/activated–carbon ratio 2:3; porous microsphere; source-specific batch conditions.	Langmuir model-derived q_max_ = 279.33 mg g^−1^ (R^2^ = 0.996).	Increased porosity and activated–carbon contribution increased Pb^2+^ uptake; ion exchange involving Ca^2+^ was supported by characterization	[32]
Chemically/ionically modified polymer-rich biomass Demethoxylated, Ca-crosslinked orange peel Composition: plant cell-wall matrix containing pectin/cellulose/hemicellulose/lignin	Mn^2+^	10 g L^−1^ sorbent; C_0_ = 100 mg L^−1^ Mn^2+^; pH 5.5 ± 0.1; 25 °C; 8 h.	Removal efficiency = 94.8 ± 0.3%; Langmuir q_max_ = 29.7 mg g^−1^.	FTIR and SEM–EDX showed abundant COO^−^/OH sites and partial replacement of residual Ca by Mn	[33]
Isolated protein biopolymer Silk fibroin powder Composition: protein matrix (semicrystalline fibroin polypeptide)	Fe(III)/total iron	Powdered fibroin; pH 6–10; optimized dose 0.25 g; 60 min.	Removal efficiency = 98%; Langmuir q_max_ = 12.82 mg g^−1^; reuse tested for 5 cycles.	Adds evidence that unmodified powdered fibroin can function as a reusable metal sorbent, although the authors’ kinetic-model interpretation should not be treated as independent proof of chemisorption	[21]
Raw polymer-rich biomass Banana peel Composition: plant cell-wall matrix containing pectin/cellulose/hemicellulose/lignin	Cd^2+^	Raw banana peel characterized by FTIR, SEM, and EDX; batch adsorption; exact source-specific conditions apply.	Kinetic endpoint: approximately 97% of equilibrium uptake reached within 10 min; absolute q not used for cross-study ranking.	Rapid uptake and spectroscopic/microscopic characterization support a heterogeneous oxygen-containing surface; capacity/selectivity remain protocol-dependent	[34]
Matched raw/depectinated biomass/isolated pectic acid Orange peel/depectinated peel/pectic acid Composition: citrus cell-wall matrix; pectin fraction varied/removed in matched materials	Cd^2+^	Matched comparison of native peel, protonated peel, depectinated peel, and extracted pectic acid.	Binding capacity: pectic acid 2.9 meq g^−1^; depectinated peel 1.7 meq g^−1^.	Directly demonstrates that pectin is important but not solely responsible for Cd binding in citrus peel	[35]
Chemically grafted polymer-rich biomass Graft-copolymer-modified orange peel Composition: plant cell-wall matrix containing pectin/cellulose/hemicellulose/lignin	Pb^2+^, Cd^2+^, Ni^2+^	Graft-copolymer-modified orange peel; pH and contact time investigated; source-specific concentration range.	Langmuir q_max_ = 476.1, 293.3, and 162.6 mg g^−1^ for Pb^2+^, Cd^2+^, and Ni^2+^, respectively.	Capacities were 4.2-, 4.6- and 16.5-fold above unmodified peel; values illustrate modification effects and must not be used as native-peel benchmarks	[36]
Chemically/ionically modified polymer-rich biomass NaOH/CaCl_2_-modified orange peel Composition: plant cell-wall matrix containing pectin/cellulose/hemicellulose/lignin	Cu^2+^, Pb^2+^, Zn^2+^	Orange peel modified with NaOH and CaCl_2_; pH and initial concentration varied in the cited study.	Langmuir q_max_ = 70.73, 209.8, and 56.18 mg g^−1^ for Cu^2+^, Pb^2+^, and Zn^2+^, respectively.	Ca^2+^ release/ion exchange at carboxyl groups was identified as a predominant mechanism; useful evidence for designed pectin-rich networks	[37]
Raw polymer-rich biomass Banana peel Composition: plant cell-wall matrix containing pectin/cellulose/hemicellulose/lignin	Cr(VI)	Untreated banana peel; industrial-wastewater application; optimum pH 2; rapid uptake reported.	Condition-specific removal = approximately 95% within 10 min; optimum pH 2.	Strong acidic optimum confirms that chromate/Cr(VI) behavior cannot be interpreted with the same cation-binding model used for Pb^2+^ or Cd^2+^	[38]
Raw polymer-rich biomass Orange peel Composition: plant cell-wall matrix containing pectin/cellulose/hemicellulose/lignin	Cr(VI)	Natural orange-peel powder; 0.315 mm fraction; acidic batch conditions; 90 min contact time.	Condition-specific removal = approximately 97.8% after 90 min; reported capacity = 4.96 mg g^−1^.	Provides a recent untreated-peel Cr(VI) benchmark with FTIR, pH_pzc_, XRD, SEM and BET characterization	[39]

**Classification note:** entries are explicitly identified as raw polymer-rich biomass, isolated biopolymer, chemically/ionically modified material, or composite network. **Metric note:** model-derived q_max_, experimentally measured uptake, percentage removal, and multicycle capacity retention are different quantities and are not treated as interchangeable. Conditions are source-specific; where a parameter is not tabulated, the cited primary study remains the controlling source. Cross-study numerical comparisons are therefore qualitative unless materials were tested under matched conditions.

**Table 2:** Metal-specific evidence, critical interpretation, and implications for biosorbent design and optional solid-phase sensing.

Target Ion	Most relevant Evidence in Selected Matrices	Critical Interpretation	Optional Analytical Prospect	Refs.
Pb^2+^	Untreated banana/orange peel provide comparable Pb uptake in the Annadurai study; low-esterified pectin binds Pb more strongly; purified pectin studies frequently place Pb among the highest-affinity ions.	Strongest and most internally consistent sorption case for carboxylate-rich matrices, but absolute capacities remain preparation-dependent.	Xylenol orange immobilized on silica enabled solid-phase diffuse-reflectance determination of Pb(II) with a detection limit of 2 μg L^−1^, demonstrating the feasibility of solid-phase optical detection, although the analytical response was reagent-mediated rather than arising from the natural polymer itself.	[2,31,41,42,47]
Cd^2+^	Banana peel shows rapid Cd uptake; pectic acid binds more Cd than depectinated orange peel; Ca-pectin gels also sorb Cd.	Strong evidence for a pectin contribution, but selectivity sequences from purified pectins do not place Cd consistently among the preferred ions.	No validated reagent-free diffuse-reflectance method was identified for the four target matrices.	[5,29,34,35,41,42]
Cr(VI)	Untreated banana and orange peels adsorb Cr(VI) most effectively under acidic conditions; acid activation and grafting can increase performance.	Must be discussed through chromate/dichromate speciation and surface protonation rather than divalent-cation mechanisms.	Cr(III), not Cr(VI), has been quantified on EBT-modified fibroin by Kubelka-Munk reflectance; the method is reagent-mediated.	[38,39,44,45,48]
Cu^2+^	Raw peels show moderate uptake; purified pectins and Ca-pectin gels often show strong Cu affinity; modified pectin-rich matrices can increase capacity.	One of the better candidates for structure-binding studies; disagreement between raw peel and isolated pectin illustrates matrix accessibility effects.	PAN/filter-paper reflectance gives a clear Cu signal but requires chromogenic ligand and masking of multiple interfering metals.	[2,5,37,41,42,49]
Ni^2+^	Raw banana/orange peel show moderate uptake; pectin affinity is source-dependent; grafted orange peel can strongly increase capacity.	No universal Ni-selective pectin behavior is supported; polymer source and modification dominate.	Ni-DMG colorimetric SPE demonstrates rapid diffuse-reflectance quantification, but selectivity comes from DMG.	[2,36,41,42,50]
Mn^2+^	Ca-crosslinked modified orange peel reached 29.7 mg g^−1^ and 94.8% removal under optimized high-concentration conditions.	High uptake belongs to an engineered matrix and does not establish trace-level analytical sensitivity.	Nitroso-R-salt/polyacrylonitrile fiber gives a 0.003 mg L^−1^ LOD; this is a useful polymer-supported benchmark but not direct orange-peel sensing.	[33,51]
Co^2+^	Untreated peels show relatively low uptake; some pectin preparations bind Co more strongly than others.	Lower priority as a primary target because affinity is inconsistent and matrix dependent.	No validated reagent-free reflectance method using the four selected matrices was identified.	[2,42]
Zn^2+^	Moderate uptake by untreated peels; purified-pectin selectivity varies and is sensitive to ionic strength and polymer source.	Sorption is established, but the evidence does not identify a uniquely favorable polymer-Zn interaction.	No convincing reagent-free optical quantification on the four target matrices was identified.	[2,37,41,42]

**Table 3:** Source-traceable examples relevant to quantitative diffuse-reflectance analysis of polymeric and metal-sensing solid phases.

Solid Phase	Analyte/Property Measured	Measurement Principle and Conditions	Reported Analytical Performance	Relevance to the Present Review	Ref.
Crosslinked poly(acrylic acid), Carbopol 974P NF	Degree of carboxylic-acid neutralization	DRIFT; carboxylic acid band at 1695 cm^−1^ and carboxylate band at 1570 cm^−1^; Kubelka–Munk treatment	Linear calibration over 0–100% neutralization	Demonstrates quantitative monitoring of carboxyl-group ionization directly in a crosslinked polymer	[59]
Commercial pectins	Degree of esterification	DRIFTS; ester C=O band centered at approximately 1756 cm^−1^	Highest reported correlation R^2^ = 0.822 for ester-band area; source-dependent spectral variation observed	Establishes direct quantitative analysis of pectin chemistry in the solid state	[9]
Commercial pectins	Degree of esterification	DRIFTS using ester-carboxyl/total-carboxyl peak-area ratio	R^2^ = 0.97 for the calibration relationship	Demonstrates improved quantitative calibration through internal spectral normalization	[10]
Xylenol-orange-modified silica	Pb^2+^	Solid-phase reagent; Pb^2+^ preconcentration followed by diffuse-reflectance spectroscopic detection; pH 4.5–5.5	Detection limit 2 µg L^−1^; applied to natural water and food samples	Demonstrates trace-metal determination by an immobilized chromogenic reagent on a solid support	[47]
Eriochrome Black T immobilized on silk fibroin	Cr^3+^	Sorption–spectrophotometric method; reflectance spectra recorded on the solid phase and evaluated using Kubelka–Munk coordinates; optimum pH 8; analytical wavelength 618 nm	LOD 1.2 µg L^−1^; linear Cr(III) amount range 5–50 µg, R^2^ = 0.9991; analysis time 10 min; relative errors ≤ 6% in industrial wastewater	Direct evidence that silk fibroin can serve as a polymeric carrier for solid-phase reflectance determination when a chromogenic reagent is immobilized	[48]
Colorimetric SPE membrane	I^−^, I_2_, Ag(I), Cu(II), Ni(II), Fe(III), Cr(VI)	SPE onto reagent-containing membrane followed by handheld diffuse-reflectance measurement	Rapid concentration/measurement cycle of approximately 1 min for the platform; analyte ranges were ion-specific	Establishes integrated solid-phase preconcentration + reflectance as a general analytical architecture, but signal is reagent-mediated	[58]
Filter paper + PAN	Cu^2+^	Spot-test complexation with 1-(2-pyridylazo)-2-naphthol; reflectance at 559 nm	R^2^ = 0.9982; LOD 0.12 mg L^−1^; LOQ 0.47 mg L^−1^; interferences reduced with masking agents	Useful benchmark for Cu optical feasibility and for the selectivity problem created by other transition-metal ions	[49]
DMG/filter support + extraction membrane	Ni^2+^	Ni-dimethylglyoxime complex collected on membrane and quantified by diffuse reflectance	Working range 0.50–5.0 ppm; approximately 40 s analysis in the reported C-SPE format	Shows rapid Ni reflectance analysis is possible when molecular recognition is supplied by DMG	[50]
Nitroso-R-salt immobilized on modified polyacrylonitrile fiber	Mn^2+^	Sorption-spectroscopic measurement; optimum pH 3–4; γ= 500 nm	Linear range 5–50 mg L^−1^; LOD 0.003 mg L^−1^; real-soil results agreed with ICP-MS (R^2^ = 0.9979)	Recent benchmark for polymer-supported Mn sensing; high sensitivity derives from immobilized chromogenic chemistry rather than the carrier polymer alone	[51]

**Table 4:** Comparative regeneration and reuse evidence for the selected natural-polymer biosorbents.

Material/Form	Target Metal(s)	Desorption/Regeneration Protocol	Cycles	Quantitative Reuse Outcome and Stability Information	Ref.
Silk fibroin extracted from *Bombyx mori*	Pb^2+^, CrO_4_^2−^, Cu^2+^, Co^2+^	EDTA stripping followed by reuse	10	No measurable loss of sorption capacity over 10 cycles; mass loss and detailed post-cycle structural metrics were not reported in the review dataset.	[7]
Silk fibroin powder	Fe species	Reuse protocol reported by source; exact eluent details should be taken from the primary article	5	Only a marginal decrease in adsorption efficiency over 5 cycles; exact percentage retention and mass loss not reported in the review dataset.	[21]
Demethylated Ca-crosslinked sugar-beet pectin xerogel	Cd^2+^, Pb^2+^, Cu^2+^	0.1 M HNO_3_ desorption; 1 M CaCl_2_ regeneration	9	Metal uptake was maintained or increased after CaCl_2_ regeneration; the primary study reported approximately 20% biomass loss over successive reuse, showing why capacity retention alone is insufficient.	[26]
Chemically modified orange peel	Cd^2+^, Cu^2+^, Pb^2+^	Acid elution; adsorption and desorption performed as a closed-loop cycle	4	Adsorption capacity was regained after four adsorption–elution cycles and metal adsorption efficiency remained around 90%.	[43]
Graft-copolymer-modified orange peel	Pb^2+^, Cd^2+^, Ni^2+^	0.05 M HCl desorption	Single desorption demonstration	Effective desorption was reported, but multicycle capacity retention, structural stability, and mass loss were not established; therefore desorption should not be equated with validated regeneration.	[36]

**Table 5:** Practical and sustainability criteria for development of the selected waste-derived biosorbents.

Criterion	What Should Be Reported	Critical Interpretation	Ref.
Precursor processing	Washing, drying, milling/sieving, extraction or fibroin regeneration conditions	Renewable origin does not capture the burden of standardizing the precursor	[60,63]
Chemical modification	Reagent identity/amount, crosslinker, pH treatment, grafting or demethylation conditions	Modification should be justified by a reproducible gain in performance, not capacity alone	[62,63]
Regeneration	Eluent, volume, desorption efficiency, capacity retention and structural stability over cycles	Desorption is not equivalent to complete regeneration of polymer function	[7,26,61]
Secondary waste	Metal concentration in eluate and fate of spent solid	Metal-loaded biomass should not be treated as ordinary biodegradable waste without safe recovery/disposal	[61]
Selectivity and matrix effects	Competing ions, ionic strength, natural/industrial water tests	Single-metal capacity does not establish practical performance	[41,42,46]
Greenness assessment	Measured reagent, energy, water and waste data for the complete procedure	AGREE/GAPI-type scoring is meaningful only after the protocol is experimentally defined	[8,64,65]
Practicality	Equilibration time, throughput, reuse, material reproducibility, instrumentation	Environmental benefit must remain compatible with functional performance	[66,67]

**Table 6:** Research gaps and recommended experiments for development of the selected waste-derived biosorbents.

Research Gap	Why It Matters	Recommended Experiment or Reporting Requirement	Most Relevant Platform(s)	Ref.
Uncontrolled precursor variability	Batch-to-batch composition can alter functional-group density, sorption and reproducibility	Record biological source, processing history, moisture, drying, particle size and representative chemical characterization for every precursor batch	Banana peel; orange peel; waste-derived pectin	[4,13,22,23]
Raw and modified materials often compared across unrelated studies	Apparent modification benefit may reflect different experimental conditions rather than material chemistry	Test untreated and modified material from the same precursor batch under identical conditions	Banana peel; orange peel; pectin	[33,35,36,37,43]
Pectin contribution within whole peel remains incompletely separated from other polymers	Whole peel also contains cellulose, hemicellulose and lignin	Compare whole peel, depectinated peel and extracted pectin where feasible	Orange peel; banana peel	[35]
Degree of pectin esterification frequently unreported	Free carboxylate density strongly affects divalent–metal interaction	Measure DE and compare native versus controlled de-esterified material	Banana/orange-derived pectin	[4,31]
Mechanisms commonly inferred from kinetic/isotherm fitting	Mathematical fit does not uniquely identify molecular mechanism	Combine sorption with FTIR, SEM–EDX/XPS where available, ion-release and surface-charge evidence	All four platforms	[5,33,35,37,40,74]
Linearized isotherm models can bias fitted parameters	Heterogeneous natural materials may contain multiple site classes	Prefer nonlinear regression; report residuals, uncertainties and model-selection criteria	All four platforms	[40,73,74]
Excessive reliance on single-metal synthetic solutions	Real waters contain competing metal and major ions	Progress from single-ion to binary, multicomponent and real-water experiments	All four platforms	[41,42,68,70]
Capacity often measured only at high concentrations	High q_max_ does not guarantee effective low-concentration polishing	Include environmentally relevant low-concentration experiments and report residual C_e_	All four platforms	[68,70,71,72]
Batch data dominate the literature	Practical operation requires dynamic mass-transfer and mechanical-stability data	Develop fixed-bed studies after batch optimization; determine breakthrough and regeneration behavior	Peels; pectin beads/foams; fibroin structures	[68]
Regeneration characterized mainly by desorption percentage	Desorption does not prove restoration of material function	Monitor adsorption capacity, mass loss, structure and performance over repeated cycles	All four platforms	[7,26,33,61,69]
Fate of metal-loaded biosorbent often ignored	Improper disposal can create secondary contamination	Quantify metal recovery and define controlled end-of-life pathway	All four platforms	[61,69,75]
Optical sensing sometimes proposed before material standardization	Morphological variability can dominate reflectance response	Establish reproducible biosorbent first; then compare full spectra across controlled metal loadings	All four, particularly pectin and fibroin	[47,48,49,50,51,54,55,56,57,58,59]
Scale-up and technology readiness remain limited	Laboratory batch success does not demonstrate practical feasibility	Evaluate material preparation at larger batch size, continuous operation and real effluents	Primarily fruit-waste-derived systems	[68,70]

**Table 7:** Proposed quantitative stage-gates for progression through the experimental roadmap.

Stage	Proposed Quantitative Go/No-Go Gate	Decision If Gate Is Not Met	Evidence/Rationale
I. Precursor standardization	Use ≥ 3 independent precursor batches; coefficient of variation (CV) ≤ 10% for moisture and at least one composition/structure proxy, with a defined particle-size fraction or geometry.	Improve sampling, washing, drying, milling, or storage before material comparison.	Precursor variability discussed in Section 7.1 and Section 7.2.
II. Baseline characterization	Demonstrate reproducible baseline characterization with CV ≤ 10% for quantitative descriptors and at least two independent descriptors relevant to the proposed mechanism (e.g., FTIR plus surface charge, SEM–EDX, swelling, BET, or XRD as appropriate).	Do not attribute performance differences to modification until the starting material is reproducible.	Mechanistic guidance in Section 3.4 and Section 7.3.
III. Minimal rational modification	Advance a modification only if a prespecified primary metric improves by ≥20% versus a matched untreated control and the recovered material mass or mechanical integrity decreases by ≤10%.	Reject or simplify the modification; reassess whether added processing is justified.	Matched-control principle in Section 7.2.
IV. Single-metal screening	For the selected application concentration, require replicate uptake/removal CV ≤ 10% and either ≥ 80% target removal or an equilibrium residual concentration below the predefined application target.	Re-optimize pH/dose or discontinue that material–metal pair.	Separates reproducibility from nominal q_max_.
V. Optimization and mechanism	Use nonlinear fitting with reported parameter uncertainty; normalized residual error ≤ 10% for the selected descriptive model and ≥2 independent experimental lines of evidence for any mechanistic claim.	Do not assign a molecular mechanism; collect additional characterization or use a descriptive interpretation only.	[40,73,74]
VI. Mixed-ion selectivity	Maintain ≥ 70% of single-ion uptake (Rcomp ≥ 70%) in the defined competitive matrix; when selectivity is claimed require αtarget/competitor > 1. Use ≥ 3 replicate tests and report uncertainty for K_d_ or α.	Reassess target/competitor chemistry, pH, modification, or intended separation claim.	Section 7.4; [41,42,68].
VII. Regeneration and multicycle performance	Retain ≥80% of initial adsorption performance after ≥5 cycles, with cumulative dry-mass loss ≤ 10% and no systematic deterioration in the selected structural/chemical stability marker.	Revise eluent/regeneration chemistry or reject the material for repeated use.	[7,21,26,43,61,69]
VIII. Real-water validation	In a documented real-water matrix, maintain ≥ 70% of the uptake observed in the matched synthetic matrix, meet the predefined residual target-metal concentration, and obtain replicate performance CV ≤ 15%. Report major ions, dissolved organic matter where relevant, and any pretreatment.	Reassess matrix pretreatment, dose, contact time, selectivity, or the intended application; do not generalize synthetic-water performance to real water.	Section 7.4 and Section 7.5; [68,69,70,71,72].
IX. Continuous-flow evaluation	Obtain ≥ 3 reproducible breakthrough runs (CV ≤ 15% for dynamic capacity) and a dynamic capacity ≥ 70% of the relevant batch equilibrium uptake under comparable feed conditions; no progressive loss of bed integrity.	Modify particle/monolith geometry, residence time, or packing strategy before scale-up.	[68,74]
X. Optional optical sensing	LOD must be below the predefined application threshold; calibration and independent validation should give repeatability RSD ≤ 10%, between-batch RSD ≤ 15%, and matrix-spike recovery 80–120%, with no >10% loss of response after regeneration.	Keep the material as a sorbent only or redesign the optical format; do not claim a validated sensing function.	Section 5.6 and Section 5.7; [47,48,49,50,51,58,76].

## Data Availability

No new data were created or analyzed in this study. Data sharing is not applicable to this article.

## Abbreviations

The following abbreviations are used in this manuscript:

AAS	atomic absorption spectroscopy;
AGREE	Analytical GREEnness Metric Approach;
AGREEprep	Analytical Greenness Metric for Sample Preparation;
BAGI	Blue Applicability Grade Index;
BET	Brunauer–Emmett–Teller;
ComplexGAPI	Complex Green Analytical Procedure Index;
DMG	dimethylglyoxime;
DRIFTS	diffuse-reflectance infrared Fourier transform spectroscopy;
EBT	Eriochrome Black T;
EDTA	ethylenediaminetetraacetic acid;
FAAS	flame atomic absorption spectroscopy;
FTIR	Fourier transform infrared spectroscopy;
GAPI	Green Analytical Procedure Index;
ICP-MS	inductively coupled plasma mass spectrometry;
ICP-OES	inductively coupled plasma optical emission spectrometry;
LOD	limit of detection;
LOQ	limit of quantification;
PAN	polyacrylonitrile;
PEI	polyethyleneimine;
SEM	scanning electron microscopy;
SEM–EDX	scanning electron microscopy coupled with energy-dispersive X-ray spectroscopy;
XPS	X-ray photoelectron spectroscopy;
XRD	X-ray diffraction.
